# 52-Week Efficacy and Safety of Telbivudine with Conditional Tenofovir Intensification at Week 24 in HBeAg-Positive Chronic Hepatitis B

**DOI:** 10.1371/journal.pone.0054279

**Published:** 2013-02-04

**Authors:** Teerha Piratvisuth, Piyawat Komolmit, Tawesak Tanwandee, Wattana Sukeepaisarnjaroen, Henry L. Y. Chan, Mário G. Pessôa, Eduardo Fassio, Suzane K. Ono, Fernando Bessone, Jorge Daruich, Stefan Zeuzem, Hugo Cheinquer, Rashidkhan Pathan, Yuhong Dong, Aldo Trylesinski

**Affiliations:** 1 NKC Institute of Gastroenterology and Hepatology, Prince of Songkla University, Songklanagarind Hospital, Hat-Yai, Songkhla, Thailand; 2 Division of Gastroenterology, Department of Medicine, Chulalongkorn University, Bangkok, Thailand; 3 Siriraj Hospital, Bangkok, Thailand; 4 Department of Medicine, Srinagarind Hospital, Khon Kaen University, Khon Kaen, Thailand; 5 Department of Medicine and Therapeutics, The Chinese University of Hong Kong, Hong Kong, Hong Kong S.A.R; 6 Hospital das Clinicas da Faculdade de Medicina da Universidade de São Paulo, São Paulo, Brazil; 7 Hospital Nacional Prof. Alejandro Posadas, Buenos Aires, Argentina; 8 Departamento de Gastroenterologia da Faculdade de Medicina da Universidade de São Paulo, São Paulo, Brazil; 9 Catedra de Clınica Medica, Universidad Nacional de Rosario, Rosario, Antigua and Barbuda; 10 Hospital de Clínicas San Martín, University of Buenos Aires, Buenos Aires, Argentina; 11 Klinikum der Johann Wolfgang Goethe-Universitaet, Frankfurt am Main, Germany; 12 Gastroenterology Division, Hospital de Clínicas de Porto Alegre, Universidade Federal do Rio Grande do Sul (UFRGS), Porto Alegre, Brazil; 13 Novartis Pharmaceuticals, East Hanover, New Jersey, United States of America; 14 Novartis Pharma AG, Basel, Switzerland; Asociacion Civil Impacta Salud y Educacion, Peru

## Abstract

**Background and Aims:**

The Roadmap concept is a therapeutic framework in chronic hepatitis B for the intensification of nucleoside analogue monotherapy based on early virologic response. The efficacy and safety of this approach applied to telbivudine treatment has not been investigated.

**Methods:**

A multinational, phase IV, single-arm open-label study (ClinicalTrials.gov ID NCT00651209) was undertaken in HBeAg-positive, nucleoside-naive adult patients with chronic hepatitis B. Patients received telbivudine (600 mg once-daily) for 24 weeks, after which those with undetectable serum HBV DNA (<300 copies/mL) continued to receive telbivudine alone while those with detectable DNA received telbivudine plus tenofovir (300 mg once-daily). Outcomes were assessed at Week 52.

**Results:**

105 patients commenced telbivudine monotherapy, of whom 100 were included in the efficacy analysis. Fifty-five (55%) had undetectable HBV DNA at Week 24 and continued telbivudine monotherapy; 45 (45%) received tenofovir intensification. At Week 52, the overall proportion of undetectable HBV DNA was 93% (93/100) by last-observation-carried-forward analysis (100% monotherapy group, 84% intensification group) and no virologic breakthroughs had occurred. ALT normalization occurred in 77% (87% monotherapy, 64% intensification), HBeAg clearance in 43% (65% monotherapy, 16% intensification), and HBeAg seroconversion in 39% (62% monotherapy, 11% intensification). Six patients had HBsAg clearance. Myalgia was more common in the monotherapy group (19% versus 7%). No decrease in the mean glomerular filtration rate occurred in either treatment group at Week 52.

**Conclusions:**

Telbivudine therapy with tenofovir intensification at Week 24, where indicated by the Roadmap strategy, appears effective and well tolerated for the treatment of chronic hepatitis B.

**Trial Registration:**

ClinicalTrials.gov NCT00651209

## Introduction

There are approximately 400 million people worldwide who are chronically infected with hepatitis B virus (HBV), of whom 75% live in the Asia-Pacific region. Chronic hepatitis B results in liver disease progressing to cirrhosis and hepatocellular carcinoma (HCC) and is responsible for approximately one million liver-related deaths per annum [1].

Treatment of HBV involves finite administration of pegylated or unpegylated interferon alfa, or indefinite administration of anti-HBV nucleoside/nucleotide analogues. Five such analogues are currently available. Lamivudine, a deoxycytidine analogue, was the first nucleoside approved for use in HBV and lamivudine monotherapy remains common despite high rates of treatment-emergent drug resistance [2]. Entecavir is a deoxyguanosine analogue with a high genetic barrier to resistance in treatment-naive patients [3]. However, lamivudine resistance predisposes HBV to subsequent entecavir resistance [4]. Telbivudine is an L-deoxythymidine analogue with superior efficacy to lamivudine [5] but a similar resistance profile [6]. Finally, the nucleotides adefovir and tenofovir are both acyclic mimetics of deoxyadenosine monophosphate which retain activity against lamivudine- and telbivudine-resistant HBV [6]. However, adefovir is associated with dose-dependent nephrotoxicity which restricts its dosing to 10 mg daily [7], at which dose it demonstrates inferior virologic efficacy to the other agents [8]–[10]. There are also concerns about the long-term safety of tenofovir, which is associated with significant loss of renal function in HIV treatment [11].

HBV viral replication is a key driver for disease progression and is associated with the development of cirrhosis and HCC [12]. The initial goal of treatment is to suppress viral replication; thereafter, sustained (on-treatment) or maintained (off-treatment) suppression of circulating HBV DNA is associated with improved serological responses and long-term outcomes [13], [14]. The emergence of drug-resistant HBV results in breakthrough viremia leading to hepatitis and liver disease progression. To ensure good long-term outcomes, the conservation of HBV DNA suppression is essential.

Early virologic response, particularly at Week 24, is associated with better long-term outcomes in chronic HBV, while detectable HBV DNA at Week 24 is associated with a higher incidence of on-therapy drug resistance [14], [15]. This predictive association has lead an international group of experts to propose the so-called “Roadmap” concept – a therapeutic algorithm for the conditional intensification of nucleoside monotherapy based on early virologic response [16]. In the Roadmap, monotherapy is continued if plasma virus is undetectable (HBV DNA <300 copies/mL) at Week 24; while for those with detectable HBV DNA defined options exist for either intensification or continued monotherapy. The Roadmap principle is widely accepted in clinical practice [17], but has yet to be prospectively evaluated. In this study, we sought to confirm prospectively the clinical utility of the Roadmap by investigating whether the conditional intensification of telbivudine monotherapy with tenofovir, when indicated by the algorithm, results in effective virologic suppression in nucleoside-naive, HBeAg-positive patients with chronic hepatitis B. We present 52-week primary efficacy and safety data.

## Materials and Methods

The protocol for this trial and supporting CONSORT checklist are available as supporting information; see Checklist S1 and Protocol S1.

### Ethics Statement

Written informed consent was obtained and eligibility assessed at a screening visit up to 6 weeks before the first dose of telbivudine. The study was approved by the institutional review boards/independent ethics committees of each study center and was conducted in compliance with the principles of the Declaration of Helsinki and in compliance with all International Conference on Harmonization Good Clinical Practice Guidelines and local regulatory requirements.

### Patients

This study (ClinicalTrials.gov ID NCT00651209) had a multinational, single-arm, open-label design. Male and female adults (≥18 years) were recruited between April 2008 and September 2009 from 17 clinical centers in Argentina (n = 3), Brazil (4), China [Hong Kong] (2), Germany (4) and Thailand (4). Major inclusion criteria were: documented chronic hepatitis B with detectable HBsAg at screening and for at least 6 months prior; HBeAg-positive (HBeAg+) and HBeAb-negative at screening; serum HBV DNA ≥5 log_10_ copies/mL by COBAS Amplicor HBV Monitor^®^ assay (Roche Molecular Systems Inc., Pleasanton, California); screening alanine aminotransferase (ALT) between 1.3× and 10× the upper limit of normal (ULN) with evidence of chronic liver inflammation (≥2 elevated ALT or aspartate aminotransferase values over at least 6 months). Exclusion criteria included: co-infection with hepatitis C virus, hepatitis D virus or HIV; hepatic decompensation; any prior nucleoside treatment or interferon/immunomodulator treatment in the 6 months before screening, or chronic renal insufficiency or serum creatinine clearance below 50 mL/min.

### Study Design

**Figure 1:**
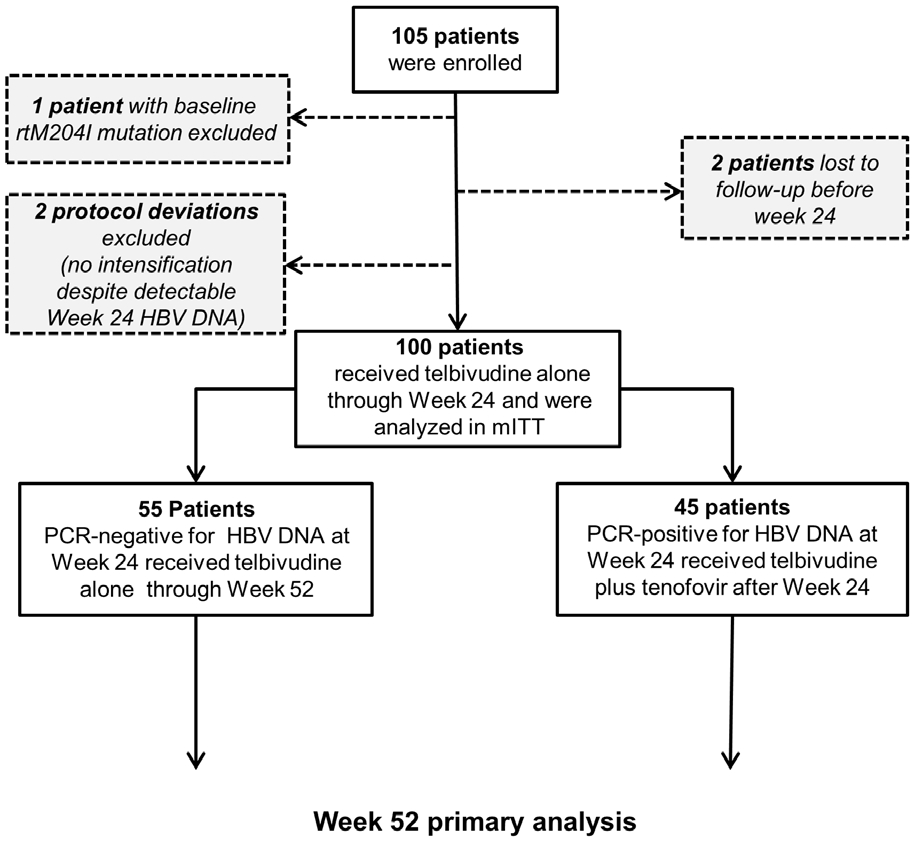
Patient disposition.

**Figure 2:**
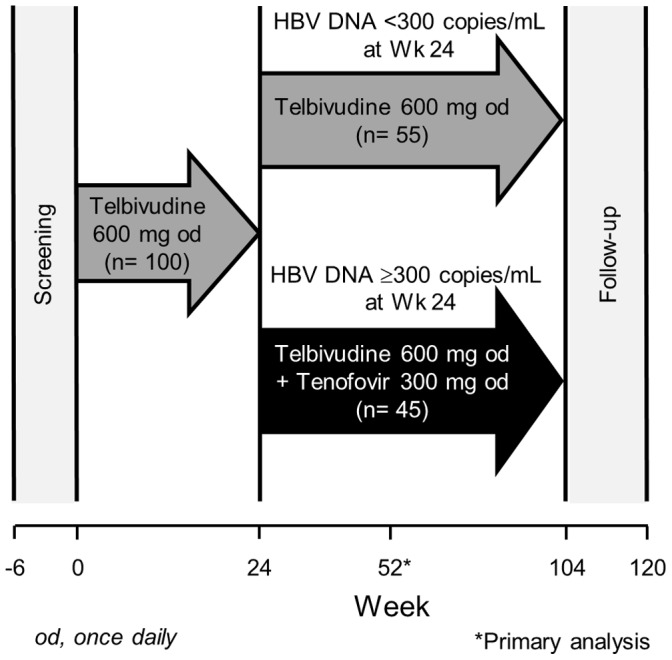
Study design.

Patient disposition is shown in Figure 1 and the study design in Figure 2. Total treatment period is 104 weeks with the primary analysis at 52 weeks. Planned study visits occurred at Weeks 2, 4, 8, 12, 16, 24, 26, 30, 40, 48, and 52. All patients received oral telbivudine (600 mg once daily) for the first 24 weeks. At Week 26, patients with detectable HBV DNA at Week 24 (≥300 copies/mL by COBAS Amplicor) received tenofovir disoproxil fumarate (300 mg once daily) in addition to telbivudine throughout the remaining time on study. Patients with undetectable HBV DNA (<300 copies/mL) at Week 24 continued to receive telbivudine monotherapy.

### Efficacy and Safety Analyses

The primary efficacy endpoint was the proportion of patients with undetectable HBV DNA at Week 52. Secondary endpoints included the rate of virological breakthrough; HBV DNA reductions from baseline and proportions with undetectable HBV DNA at each study visit; ALT normalization rates at Weeks 24 and 52, and rates of HBeAg and HBsAg loss and seroconversion at Week 52.

Clinical and laboratory adverse events were graded according to pre-specified criteria and reported relationship to study drug was at investigator discretion. ALT flares were defined according to AASLD criteria [18]. Glomerular filtration rates (GFR) at each visit were estimated using both the Cockcroft-Gault [19] and the Modification Of Diet In Renal Disease Study (MDRD) equations [20], [21].

### Statistical Analysis

The primary efficacy endpoint was analyzed as a binary variable using last-observation-carried-forward (LOCF) imputation. The primary endpoint and all binary secondary endpoints were summarized at each visit, with the 95% confidence interval calculated using the normal approximation method for binomial proportion. Differences in baseline demographics and disease characteristics between patients who remained on telbivudine monotherapy through Week 52 and those who added tenofovir after Week 24 were assessed for significance using Fisher’s exact test for categorical variables and two-sided t-tests for continuous variables. Changes from baseline in GFR at Week 52 were assessed for significance using two-sided, paired t-tests. Statistical analyses were carried out using SAS ver 1.3 (SAS Institute, Inc. Carey, NC, USA).

The full intention-to-treat (ITT) population comprised all patients who received at least one dose of telbivudine. As this was a study of a specific therapeutic algorithm, a per-protocol population was derived for efficacy assessments – i.e. the efficacy population – which excluded one patient with a confirmed baseline rtM204I resistance mutation to telbivudine/lamivudine, 2 patients lost to follow-up before Week 24, and 2 protocol violators who did not receive tenofovir despite detectable Week 24 HBV DNA. The safety population for adverse event monitoring comprised the full ITT population as defined.

This was a single-arm study, hence the sample size was not based on power for statistical comparison between treatment groups. The primary objective was to demonstrate antiviral efficacy by estimating the proportion of patients with HBV DNA <300 copies/mL at Week 52.

Based on the pivotal phase III GLOBE study (NCT00057265) [15], approximately 44% of patients treated with telbivudine were anticipated to have HBV DNA <300 copies/mL at Week 24, of whom 95% would still be <300 copies/mL at Week 52. By contrast, it was assumed from GLOBE data that only 34% of patients with HBV DNA of 300 copies/mL or above at Week 24 would fall to below 300 copies/mL by Week 52 on telbivudine monotherapy, giving an overall proportion of 60% with <300 copies/mL at Week 52. It was assumed that adding tenofovir at Week 24 for those with ≥300 HBV DNA copies/mL would increase the proportion who subsequently fell to <300 copies/mL at Week 52 to at least 50%. With 100 patients, and under these assumptions, the estimated 95% confidence interval was 70% ±9%, which gave over 95% chance to show a higher rate of HBV DNA <300 copies/mL over telbivudine mono therapy in GLOBE and also provided a reasonably accurate estimate. However, it remains important to note that the two groups after Week 24– telbivudine and telbivudine plus tenofovir – were not randomized and hence statistical comparisons are limited. In particular, the lack of randomization, and confounding by Week 24 response to telbivudine, precludes efficacy comparison between the telbivudine and telbivudine plus tenofovir groups.

## Results

### Patient Disposition

The efficacy population comprised 100 patients and the safety population 105 patients (Figure 2).

Patient demographics and baseline characteristics are shown in Table 1, stratified according to treatment after Week 24. Compared with those who remained on telbivudine monotherapy, a higher proportion of intensification patients had baseline HBV DNA ≥9 log_10_ copies/mL (73.3% versus 36.4% of those remaining on monotherapy; P<0.001). Mean baseline ALT was also higher in those who remained on monotherapy (167.2 U/L versus 93.2 U/L; P = 0.0045). Other characteristics were broadly similar between those who did and did not receive intensification.

**Table 1:** Demographics and baseline characteristics (efficacy population) according to post-Week 24 treatment.

Characteristic		Telbivudine	Telbivudine+tenofovir	*P value*	Overall
N		55	45		100
Age, mean (SD) y		37 (10.4)	40 (15.0)	0.2394	38 (12.7)
Male, n (%)		37 (67)	30 (67)	1.0000	67 (67)
Weight, mean (SD) kg		69.7 (15.0)	65.5 (13.5)	0.1419	67.8 (14.4)
Race, n (%)	Caucasian	11 (20)	16 (36)	0.0550	27 (27)
	Black	0	1 (2)		1 (1)
	Asian	41 (75)	28 (62)		69 (69)
	Other	3 (6)	0		3 (3)
HBV genotype, n (%)	A	6 (11)	8 (18)	0.2192	14 (14)
	B	5 (9)	6 (13)		11 (11)
	C	35 (64)	22 (49)		57 (57)
	D	1 (2)	5 (11)		6 (6)
	F	7 (13)	3 (7)		10 (10)
	Intermediate	1 (2)	1 (2)		2 (2)
Serum ALT, mean (SD) U/L		167.2 (162.2)	93.2 (57.8)	**0.0045**	133.9 (131.2)
Serum HBV DNA (copies/mL), n (%)	5– <6 log_10_	4 (7)	1 (2)		5 (5)
	6– <7 log_10_	7 (13)	1 (2)		8 (8)
	7– <8 log_10_	11 (20)	4 (9)		15 (15)
	8– <9 log_10_	13 (24)	6 (13)		19 (19)
	≥9 log_10_	20 (36)	33 (73)	**<0.001**	53 (53)
GFR, mean (SD) mL/min/1.73 m^2^ by MDRD		93.4 (15.1)	92.1 (18.5)	0.6873	92.8 (16.6)

doi:10.1371/journal.pone.0054279.t001

A total of 99/100 patients in the efficacy population (99%) completed Week 52. There was one discontinuation in the telbivudine plus tenofovir group for loss to follow-up after Week 30.

### Efficacy

At Week 24, 55 of 100 patients (55%) in the efficacy population had undetectable HBV DNA and continued to receive monotherapy. All of these 55 patients remained undetectable at Week 52 on telbivudine monotherapy. The remaining 45 patients (45%) received telbivudine plus tenofovir after Week 24, of whom 38 (84.4%) had undetectable DNA at Week 52. Of these 45 patients, 12 had baseline HBV DNA <9 log_10_ copies/mL (of whom 3 also had baseline ALT ≥2×ULN) and 33 had ≥9 log_10_ copies/mL_._ All (12/12) of the patients with baseline HBV DNA <9 log_10_ copies/mL, and 78.8% (26/33) of those with ≥9 log_10_ copies/mL, achieved undetectable DNA at week 52.

The overall rate of undetectable HBV DNA at Week 52 (primary endpoint) was therefore 93% (93/100) by LOCF analysis. This value was the same by a strict ITT missing = failure analysis, as one patient lost to follow-up after Week 30 had detectable HBV DNA (2.67 log) at last visit.

**Figure 3:**
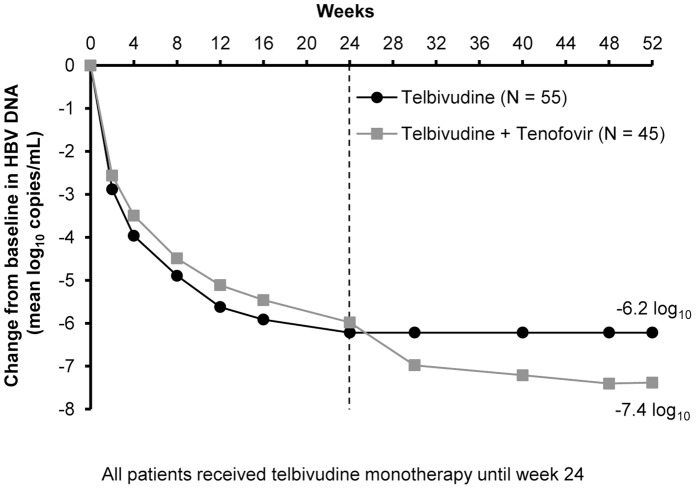
Changes from baseline in HBV DNA by post-Week 24 treatment (efficacy population).

Primary and secondary efficacy endpoints are shown in Table 2. Figure 3 shows mean changes from baseline in HBV DNA by visit for the two treatment groups. By LOCF analysis, mean reduction from baseline in HBV DNA at Week 24 was −6.2 log_10_ copies/mL in patients who continued to receive telbivudine alone, versus −6.0 log_10_ copies/mL in those who subsequently received tenofovir. The Week 24 mean reduction remained stable at −6.2 log_10_ through Week 52 in those who continued telbivudine monotherapy, while the addition of tenofovir resulted in an additional 1.4 log_10_ reduction at Week 52 in the intensification group.

**Table 2:** Results of efficacy endpoints up to Week 52 (efficacy population, LOCF).

n (%)	Efficacy endpoint	Telbivudine monotherapy (n = 55)	Telbivudine+Tenofovir (n = 45)	Overall (N = 100)
**Week 24**	HBV DNA <300 copies/mL	55/55 (100)	0/45	55/100 (55.0)
**Week 52**	HBV DNA <300 copies/mL	55/55 (100)	38/45 (84.4)	93/100 (93.0)
	Virologic breakthrough	0/55 (0)	0/45 (0)	0/100 (0)
	HBeAg loss*	36/55 (65.5)	7/44 (15.9)	43/99 (43.4)
	HBeAg seroconversion*	34/55 (61.8)	5/44 (11.4)	39/99 (39.4)
	HBsAg loss*	1/55 (1.8)	5/44 (11.4)	6/99 (6.1)
	HBsAg seroconversion*	0/55 (0)	3/44 (6.8)	3/99 (3.0)
	ALT normalization	48/55 (87.3)	29/45 (64.4)	77/100 (77.0)

* HBeAg/HBsAg loss and seroconversion were evaluated at Week 52 only without LOCF imputation. HBeAg/HBsAg data were unavailable for 1/45 patients receiving telbivudine + tenofovir.

doi:10.1371/journal.pone.0054279.t002

Overall, 43.4% of patients (43/99) with available data at Week 52 lost HBeAg and 39.4% (39/99) achieved HBeAg seroconversion. Rates of HBeAg loss and seroconversion among those who remained on monotherapy (65.5% and 61.8%, respectively) were approximately fourfold higher than among those who received intensification (15.9% and 11.4%, respectively). HBsAg clearance at Week 52 occurred in 6.1% (6/99) and HBsAg seroconversion in 3.0% (3/99). Of the six patients with HBsAg loss, one (Genotype B) was in the monotherapy group and five (3 Genotype A, 1 F;1 B) in the intensification group; four were Hispanic Caucasians and two were other races, and all had baseline HBV DNA >9 log_10_ copies/mL.

Overall, 77% of patients achieved ALT normalization at Week 52: 48/55 (87%) in the monotherapy group and 29/45 (64%) in the intensification group.

No virologic breakthrough and no genotypic resistance over 52 weeks was observed.

### Safety

Adverse events through Week 52 in the safety population are shown in Table 3. Adverse events were similar to the GLOBE study and balanced between treatment groups.

**Table 3:** Most common (≥5%) all-cause adverse events through Week 52 (safety population).

		Tenofovir intensification (n = 46)		
n (%)	Telbivudine monotherapy (n = 59)	Telbivudine onlyperiod	Tenofovir add onperiod	Overall*(N = 105)
**Total patients with any event**	**42 (71.2)**	**23 (50.0)**	**20 (43.5)**	**73 (69.5)**
Myalgia	10 (16.9)	3 (6.5)	1 (2.2)	13 (12.4)
Headache	6 (10.2)	5 (10.9)	1 (2.2)	12 (11.4)
Upper respiratory tract infection	4 (6.8)	4 (8.7)	1 (2.2)	9 (8.6)
Dyspepsia	4 (6.8)	0 (0.0)	3 (6.5)	7 (6.7)
Arthralgia	1 (1.7)	2 (4.3)	3 (6.5)	5 (4.8)
Diarrhoea	3 (5.1)	1 (2.2)	1 (2.2)	5 (4.8)
Nausea	2 (3.4)	0 (0.0)	3 (6.5)	5 (4.8)
Dizziness	3 (5.1)	1 (2.2)	0 (0.0)	4 (3.8)
Fatigue	3 (5.1)	1 (2.2)	0 (0.0)	4 (3.8)
Pain in extremity	3 (5.1)	1 (2.2)	0 (0.0)	4 (3.8)
Pyrexia	3 (5.1)	1 (2.2)	0 (0.0)	4 (3.8)
Vomiting	1 (1.7)	0 (0.0)	3 (6.5)	4 (3.8)
Nasopharyngitis	3 (5.1)	0 (0.0)	1 (2.2)	4 (3.8)
Upper abdominal pain	0 (0.0)	3 (6.5)	0 (0.0)	3 (2.9)
Cough	0 (0.0)	2 (4.3)	1 (2.2)	3 (2.9)

* Intensification patients may have had an event during both the telbivudine only and intensification periods. Overall numbers with an indicated event may therefore be less than the row total.

doi:10.1371/journal.pone.0054279.t003

There were no deaths. Five serious adverse events occurred, comprising one case each of atrial septal defect, gallbladder polyp, vascular injury and spontaneous abortion on telbivudine alone; and one foot fracture on telbivudine plus tenofovir. No event was considered treatment related.

There were no reports of myopathy, myositis, rhabdomyolysis, lactic acidosis, pancreatitis or peripheral neuropathy. One patient reported mild muscle weakness. The study treatment was interrupted and patient followed up in-study. This patient (highest creatine kinase 1866 IU/mL) did not have objective evidence of decreased muscle strength or abnormal EMG or muscle biopsy results. Myalgia occurred in 13 patients. Twelve were mild and one was moderate. Twelve resolved and one was followed up in-study. Twelve patients continued the study treatment without interruption and one discontinued. Six myalgia cases were not suspected to be related to study treatment.

Grade 3 or grade 4 creatine kinase elevations occurred in two patients in the monotherapy group (one grade 3 at Week 48 and one grade 4 at Week 49) and two intensification group patients (one grade 3 at Week 16 on telbivudine alone and another at Week 52 on telbivudine plus tenofovir).

Two patients experienced an ALT flare. Both occurred under initial telbivudine monotherapy (Weeks 4 and 8) in patients who later received intensification. Both flare patients had undetectable HBV DNA at Week 52.

**Figure 4:**
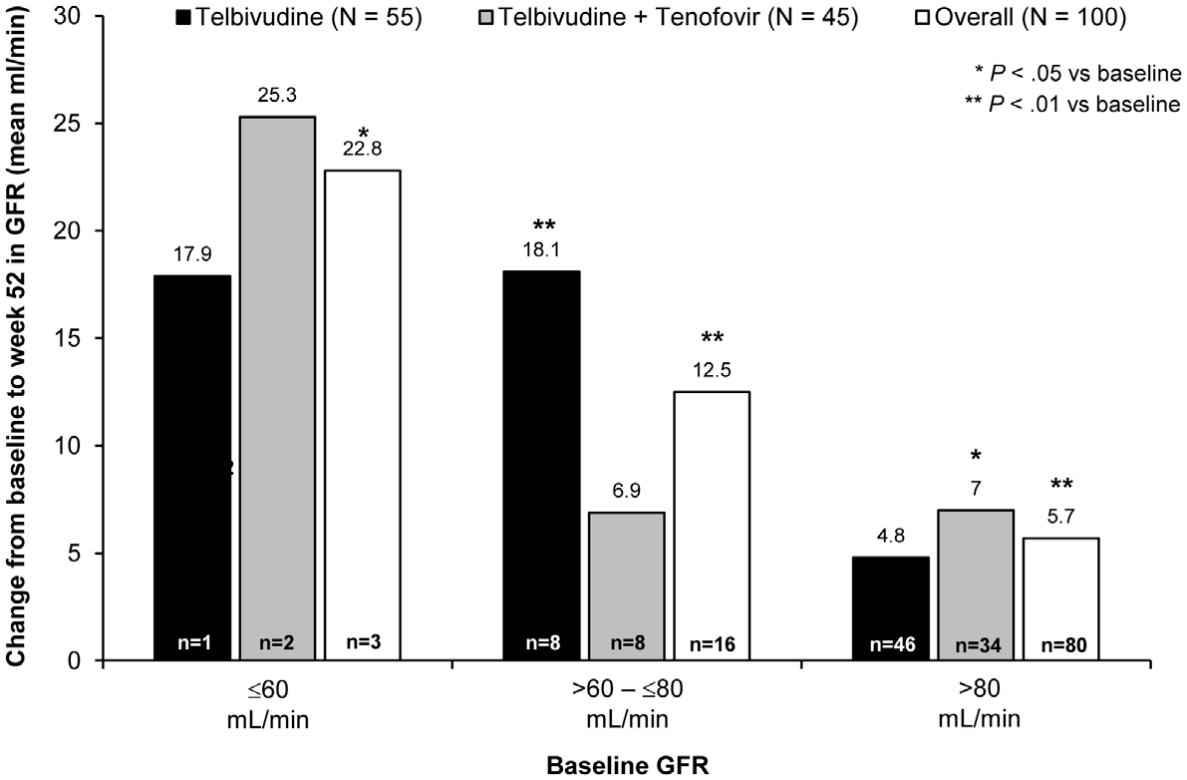
Week 52 glomerular filtration rate (MDRD) changes, by baseline rate and treatment (efficacy population).

There were no renal events other than one treatment-unrelated case of moderate renal colic. No patient reported with creatinine increase. Mean Week 52 GFR was significantly higher than baseline in both treatment groups. Overall mean GFR change to week 52 by the MDRD and the Cockcroft-Gault formulae was 6.9 mL/min and 8.3 mL/min, respectively, in the monotherapy group; and 7.4 mL/min and 6.2 mL/min, respectively, in the intensification group (P<0.01 for all comparisons). Figure 4 shows week 52 GFR changes (MDRD) stratified by baseline GFR. No significant GFR decline was noted in patients with low baseline GFR, although numbers were small.

## Discussion

In providing a framework for the conditional intensification of monotherapy, the Roadmap [16] provides a rational approach to improve long-term outcomes, while minimizing the risk of drug resistance due to continued sub-optimal monotherapy or adverse events associated with unnecessary combination treatment. Under the Roadmap, nucleosides with a low genetic barrier to resistance (e.g. lamivudine) are intensified with a non-cross-resistant second agent if any HBV replication is still measurable at Week 24. Although telbivudine is more potent than lamivudine with less on-treatment resistance [5],[15], it is closest to lamivudine in terms of the features informing Roadmap decisions; and intensification was applied to any patient with detectable Week 24 viremia. The requirement for a non-cross-resistant second agent predicates the use of adefovir or tenofovir [6], with tenofovir the better choice due to greater potency [10].

Over half the patients achieved undetectable Week 24 viremia on telbivudine alone, and, following tenofovir intensification of the remaining 45%, the overall proportion of undetectable HBV DNA was greater than 90% at Week 52. The additional reduction in HBV DNA seen following tenofovir intensification of viremic patients is presumably due to additive antiviral activity. There was no in-study comparator to estimate the treatment effect of tenofovir intensification over continued telbivudine monotherapy in viremic patients. Nor was tenofovir monotherapy investigated, or the effect of switching viremic patients to tenofovir as opposed to adding it to telbivudine. However, historical data suggest that intensification would have significantly improved Week 52 outcomes over what would have been seen had telbivudine monotherapy been continued. In GLOBE [15] a broadly similar rate of undetectable viremia at Week 24 (45%) was seen in HBeAg+ telbivudine patients to that seen here (55%), but with a substantially lower rate of undetectable DNA at Week 52 (60%). GLOBE also showed lower rates of undetectable HBV DNA, ALT normalization and HBeAg seroconversion at Week 52, and higher rates of drug resistance at Week 48, for patients with detectable Week 24 viremia [15], although the design of these analyses precludes cross-study comparison. Similar results were observed in a study of telbivudine versus lamivudine in over 300 Chinese patients [22]. Finally, in the two-year GLOBE analyses, 82% (166/203) of HBeAg+ patients with undetectable Week 24 HBV remained undetectable through two years, but only 36% (89/247) of those with detectable Week 24 DNA were undetectable at the end of Year 2 [23].

HBeAg clearance and seroconversion rates were very high (Table 2), with most (approximately 85% of cases) occurring in those with undetectable Week 24 viremia who remained on telbivudine monotherapy. Effective clearance and seroconversion of HBeAg therefore appears to be a function of early and complete virologic suppression. The 6% rate of HBsAg loss at 1 year of treatment was also substantially higher than the typically reported per-annum rates of <1% on nucleosides and approximately 3% on interferon treatment [24],[25]. The association of HBsAg response with intensification (5/6 cases of loss and all three cases of seroconversion) suggests a potential synergistic effect between tenofovir and telbivudine that merits longer-term investigation in a larger dataset.

Safety and tolerability were consistent with GLOBE, and, other than myalgia, muscle-related events were rare. Of 13 patients with myalgia, most (12/13) experienced mild events and most (12/13) resolved sponataneously.

No renal toxicity was observed after 24 weeks of tenofovir plus telbivudine. Mean GFR at week 52 was significantly higher than baseline in both the monotherapy and intensification groups. These findings are consistent with both 2-year clinical data from a study of telbivudine versus lamivudine in decompensated HBV disease [26]. Furthermore, retrospective analyses of seven studies (2500 patients) in both compensated and decompensated disease showed consistent GFR improvements on telbivudine treatment for up to 6 years compared with GFR declines on lamivudine therapy. Improvement was greatest in patients more than 50 years old and those with abnormal baseline GFR; and was not associated with baseline ascites, virologic response or reduction in Child-Pugh score [27]. GFR improvement on telbivudine stands in contrast to the declines over time observed in studies of tenofovir [28] and entecavir [29]. Interestingly, GFR modeling data from Mauss *et al.* predict a year-on-year GFR reduction of approximately 2 mL/min in untreated HBV monoinfection which is halved, but not abolished, by monotherapy with lamivudine, adefovir, entecavir or tenofovir [30]. Telbivudine was not studied in the Mauss model, and more research is needed to confirm and provide a mechanism for the apparent dissimilarity of telbivudine to the other nucleosides with respect to GFR preservation.

The Roadmap algorithm does not consider baseline HBV DNA in treatment decisions [16]. However, in this study, high baseline DNA was predictive of detectable Week 24 viremia requiring intensification. Almost three-quarters of patients who received tenofovir had baseline HBV DNA ≥9 log_10_ copies/mL. In future, baseline viremia may need to be considered in any treatment algorithm where decisions are made on the presence of detectable viremia early on therapy.

In conclusion, telbivudine with conditional tenofovir intensification according to the Roadmap algorithm was well tolerated and, over 52 weeks, resulted in very high rates of undetectable HBV DNA, ALT normalization, and HBeAg/HBsAg clearance and seroconversion in nucleoside-naive HBeAg+ patients with chronic HBV infection, along with an improvement in GFR. The Roadmap appears to be a highly effective approach to HBV treatment and 104-week data from this study are awaited.

## Supporting Information

Table S1 List of ethics committees/institutional review boards. (PDF)

Checklist S1 **CONSORT checklist.**
(DOCX)

Protocol S1 **Study protocol.**
(PDF)
