# Supplementary material for: 52-Week Efficacy and Safety of Telbivudine with Conditional Tenofovir Intensification at Week 24 in HBeAg-Positive Chronic Hepatitis B
Source: PLoS One. 2013 Feb 4;8(2):e54279. doi: 10.1371/journal.pone.0054279 (PMC3563589; doi:10.1371/journal.pone.0054279)
Supplement: Table S1 — (PDF) [file pone.0054279.s001.pdf]

Table S1. List of ethics committees/institutional review boards

Committee or board	Department/organization	Address
Comissão de Ética para Análise de Projetos de Pesquisa do HCFMUSP e FMUSP	Diretoria Clínica do Hospital das Clínicas da Faculdade de Medicina da Universidade de São Paulo	São Paulo SP<br>05403-010<br>Brazil
Institutional Review Board, Faculty of Medicine, Chulalongkorn University		Bangkok Bangkok<br>10330<br>Thailand
Siriraj Ethics Committee		Bangkok Bangkok<br>10700<br>Thailand
Ethik-Kommission des Landes Berlin	Landesamt fuer Gesundheit und Soziales	Berlin Berlin<br>10707<br>Germany
Comitê de Ética em Pesquisa HCRP e FMRP-USP	Hospital das Clínicas da Faculdade de Medicina de Ribeirão Preto da Universidade de São Paulo	Ribeirão Preto SP<br>14048-900<br>Brazil
Ethik-Kommission	Aerztekammer Hamburg	Hamburg Hamburg 22083<br>Germany
Ethik-Kommission	Medizinische Hochschule Hannover	Hannover<br>Niedersachsen<br>30625<br>Germany
The Khon Kaen University Ethics Committee For Human Research		Khon Kaen Khon Kaen 40002<br>Thailand
Ethik-Kommission	Fachbereichs Medizin der Johann Wolfgang Goethe-Universität	Frankfurt am Main<br>Hessen<br>Germany
Comissão Científica e Comissão de Pesquisa e Ética em Saúde	Grupo de Pesquisa e Pós-Graduação	Porto Alegre RS Brazil
Comité Independiente de Etica para Ensayos en Farmacología Clínica	Pte. J. E. Uriburu 774, 1st Floor	Buenos Aires Buenos Aires C1027AAP<br>Argentina
Joint The Chinese University of Hong Kong - New Territories East Cluster Clinical Research Ethics Committee		Hong Kong
Comité Independiente de Etica Hospital Provincial del Centenario	Santa Fe 3100	Rosario Santa Fe<br>2000<br>Argentina
Fundación Favaloro	Av. Belgrano 1746	Buenos Aires Buenos Aires C1093AAS<br>Argentina
Kowloon West Cluster Clinical Research Ethics Committee		Hong Kong
Ethics Committee, Faculty of Medicine, Prince of Songkla University		Songkla Songkla<br>90110<br>Thailand
