# Supplementary material for: 52-Week Efficacy and Safety of Telbivudine with Conditional Tenofovir Intensification at Week 24 in HBeAg-Positive Chronic Hepatitis B
Source: PLoS One. 2013 Feb 4;8(2):e54279. doi: 10.1371/journal.pone.0054279 (PMC3563589; doi:10.1371/journal.pone.0054279)
Supplement: Protocol S1 — (PDF) [file pone.0054279.s003.pdf]

NOVARTIS

Clinical Development & Medical Affairs

LDT600, Telbivudine (Sebivo®) tablets

Clinical Study Protocol CLDT600A2410

A single-arm, multinational, two year study evaluating the efficacy and safety of lead-in telbivudine for 24 weeks with or without tenofovir treatment intensification in adult patients with HBeAg-positive chronic hepatitis B

Authors:	Dong Yuhong, Poirier Kevin, Gysen Michele, Uddin Molla, Rashidkhan Pathan Trylesinski Aldo
Principle or Coordinating Investigator	Non applicable
Document type:	Amended Protocol Version
EUDRACT number:	2007-004845-14
Version number:	v03 clean version
Development phase:	IV
Release date:	29-Oct-2011

Property of Novartis
Confidential
May not be used, divulged, published, or otherwise disclosed
without the consent of Novartis

This document (090095af830c511d in docbase CREDI_EH) has been digitally signed with external signatures using Entrust PKI.
Signatures manifested as of 11/5/2011 6:28:07 AM, signing status at this time: Completed (1 of 1 signatures)
Approved for report publication by Poirier Kevin in East Hanover at Sat, 05 Nov 2011 02:19:57 AM EDT

Table of contents

Table of contents 2
List of tables 4
List of figures 5
List of abbreviations 6
Glossary of terms 9
Amendment 3 11
Protocol synopsis 14
1 Background 21
1.1 Chronic Hepatitis B: Disease overview 21
1.2 Current treatments for CHB 21
1.2.1 Combination Therapy with Antivirals 22
1.2.2 Telbivudine 22
1.2.3 Tenofovir 22
1.3 Drug-Drug Interaction Studies 23
2 Purpose and rationale 23
3 Objectives 24
3.1 Primary objective 24
3.2 Secondary objectives 24
3.3 Exploratory objectives 24
4 Study design 25
5 Population 26
5.1 Inclusion criteria 26
5.2 Exclusion criteria 26
6 Treatment 28
6.1 Investigational and control drugs 28
6.2 Treatment arm 28
6.3 Treatment assignment 29
6.4 Treatment blinding 29
6.5 Treating the patient 29
6.5.1 Patient numbering 29
6.5.2 Dispensing the study drug 29
6.5.3 Study drug supply, storage and tracking 29
6.5.4 Instructions for prescribing and taking the study drug 30
6.5.5 Permitted study drug dose adjustments and interruptions 31
6.5.6 Rescue medication 31

This document (090095af830c511d in docbase CREDI_EH) has been digitally signed with external signatures using Entrust PKI.
Signatures manifested as of 11/5/2011 6:28:07 AM, signing status at this time: Completed (1 of 1 signatures)
Approved for report publication by Poirier Kevin in East Hanover at Sat, 05 Nov 2011 02:19:57 AM EDT

6.5.7 Other concomitant treatment 31
6.5.8 Study drug discontinuation and premature patient withdrawal 32
6.5.9 Nephrotoxicity 33
6.5.10 Signs of hepatic decompensation 34
6.5.11 Lack of efficacy at Week 24 or thereafter (persisting ALT elevation and evidence of insufficient viral response or Virologic Breakthrough) 34
6.5.12 Emergency unblinding of treatment assignment 35
6.5.13 Study completion and post-study treatment 35
6.5.14 Early study termination 36
7 Visit schedule and assessments 36
7.1 Information to be collected on screening failures 40
7.2 Patient demographics/other baseline characteristics 40
7.3 Treatment exposure and compliance 40
7.4 Efficacy 41
7.4.1 Serum HBV DNA Assessments 41
7.4.2 Serologic Markers 41
7.4.3 ALT 41
7.5 Safety 41
7.5.1 Physical examination 41
7.5.2 Vital signs 42
7.5.3 Height and weight 42
7.5.4 Laboratory evaluations 42
7.5.5 Electrocardiogram (ECG) 42
7.5.6 Pregnancy and assessments of fertility 42
7.5.7 Unscheduled safety visits 43
7.6 Other assessments 43
8 Safety monitoring 43
8.1 Adverse events 43
8.2 Serious adverse event reporting 44
8.3 Pregnancies 45
8.4 Adverse events of special interest (AESIs) reporting 45
8.4.1 Clinical trial subjects with musculoskeletal complaints in Clinical Trial Subjects with Musculoskeletal Complaints 46
8.5 Data Monitoring Committee 47
9 Data review and database management 47
9.1 Site monitoring 47

This document (090095af830c511d in docbase CREDI_EH) has been digitally signed with external signatures using Entrust PKI.
Signatures manifested as of 11/5/2011 6:28:07 AM, signing status at this time: Completed (1 of 1 signatures)
Approved for report publication by Poirier Kevin in East Hanover at Sat, 05 Nov 2011 02:19:57 AM EDT

9.2 Data collection ... 48
9.3 Database management and quality control ... 48
10 Data analysis ... 49
10.1 Populations for analysis ... 49
10.2 Patient demographics/other baseline characteristics ... 49
10.3 Treatments (study drug, rescue medication, other concomitant therapies, compliance) ... 50
10.4 Analysis of the primary objective(s) ... 50
10.4.1 Variable ... 50
10.4.2 Statistical method of analysis ... 50
10.4.3 Handling of missing values/censoring/discontinuations ... 50
10.4.4 Supportive analyses ... 51
10.5 Analysis of secondary objectives ... 51
10.5.1 Efficacy (secondary) ... 51
10.5.2 Safety ... 53
10.6 Sample size calculation ... 53
10.7 Power for analysis of critical secondary variables ... 54
10.8 Interim analysis ... 54
11 Ethical considerations ... 54
11.1 Regulatory and ethical compliance ... 54
11.2 Informed consent procedures ... 54
11.3 Responsibilities of the investigator and IRB/IEC/REB ... 55
11.4 Publication of study protocol and results ... 55
12 Protocol adherence ... 55
12.1 Protocol Amendments ... 55
13 References: available upon request ... 57
14 Appendix 1: Clinically notable laboratory values and vital signs ... 59
15 Appendix 2: Prohibited Concurrent Medications/Permitted Concurrent Medication ... 60
16 Appendix 3: Initial Muscle Symptom Questionnaire ... 61
17 Appendix 4: Follow up Muscle Symptom Questionnaire ... 62
18 Appendix 5: Muscle Strength Testing Exam ... 63

List of tables

Table 6-1 Treatment group assignment and instructions for study drug use ... 29
Table 6-2 Signs of hepatic decompensation ... 34

This document (090095af830c511d in docbase CREDI_EH) has been digitally signed with external signatures using Entrust PKI.
Signatures manifested as of 11/5/2011 6:28:07 AM, signing status at this time: Completed (1 of 1 signatures)
Approved for report publication by Poirier Kevin in East Hanover at Sat, 05 Nov 2011 02:19:57 AM EDT

Table 6-3 ALT elevation and insufficient viral response, or Virologic Breakthrough requiring withdrawal from study....................................35
Table 7-1 Assessment schedule...38
Table 10-1 Method of Handling Missing Data for Treatment Failure, Patient Withdrawal and Intermittent Missing Data..52

List of figures

Figure 4-1 Study design..25

This document (090095af830c511d in docbase CREDI_EH) has been digitally signed with external signatures using Entrust PKI.
Signatures manifested as of 11/5/2011 6:28:07 AM, signing status at this time: Completed (1 of 1 signatures)
Approved for report publication by Poirier Kevin in East Hanover at Sat, 05 Nov 2011 02:19:57 AM EDT

List of abbreviations

ADV	Adefovir Dipivoxil (Hepsera®)
AE	Adverse Event
AFP	Alpha-fetoprotein
Alb	Albumin
ALT	Alanine Aminotransferase/Glutamic Pyruvic Transaminase/GPT
ANC	Absolute Neutrophil Count
ANCOVA	Analysis of Covariance
AST	Aspartate Aminotransferase/Glutamic Oxaloacetic Transaminase/GOT
BUN	Blood Urea Nitrogen
CHB	Chronic Hepatitis B
CI	Confidence Interval
CK	Creatine Kinase (formally creatine phosphokinase)
CPO	Country Pharma Organization
CRD	Clinical Research and Development
CRF	Case Report/Record Form
CRO	Contract Research Organization
Cr_s	Serum creatinine
CS&E	Clinical Safety and Epidemiology
DNA	Deoxyribonucleic Acid
EC	Ethics Committee
ECG	Electrocardiogram
eCRF	electronic Case Report Form
EDC	Electronic Data Capture
ETV	Entecavir (Baraclude™)
FSH	Follicle Stimulating Hormone
GCP	Good Clinical Practice
HBcAg	Hepatitis B core Antigen
HBeAb	Hepatitis B “e” Antibody
HBeAg	Hepatitis B “e” Antigen
HBsAb	Hepatitis B surface Antibody
HBsAg	Hepatitis B surface Antigen

This document (090095af830c511d in docbase CREDI_EH) has been digitally signed with external signatures using Entrust PKI.
Signatures manifested as of 11/5/2011 6:28:07 AM, signing status at this time: Completed (1 of 1 signatures)
Approved for report publication by Poirier Kevin in East Hanover at Sat, 05 Nov 2011 02:19:57 AM EDT

HBV	Hepatitis B Virus
HCC	Hepatocellular Carcinoma
β-HCG	Beta-Human Chorionic Gonadotropin
Hct	Hematocrit
HCV	Hepatitis C Virus
HDV	Hepatitis D Virus
Hgb	Hemoglobin
HIV	Human Immunodeficiency Virus
ICH	International Conference on Harmonization of Technical Requirements for Registration of Pharmaceuticals for Human Use
IEC	Independent Ethics Committee
IFN	Interferon
IRB	Institutional Review Board
ITT	Intent To Treat
IUD	Intra-Uterine Device
IIVRS	Interactive Voice Response System
LAM	Lamivudine (Epivir-HBV®)
LDT	Telbivudine (Sebivo®)
LLD	Lower Limit of Detection
LLOQ	Lower Limit of Quantification
LOCF	Last Observation Carried Forward
LFT	Liver Function Test
MedDRA	Medical Dictionary for Regulatory Activities
MHC	Major Histocompatibility Complex
NSAID	Non-Steroidal Anti-Inflammatory Drug
o.d.	Omnia die/Once a Day
PCR	Polymerase Chain Reaction
p.o.	Per os/By Mouth/Orally
pp	Per-Protocol
PT/INR	Prothrombin Time/International Normalized Ratio
REB	Research Ethics Board
SAE	Serious Adverse Event

This document (090095af830c511d in docbase CREDI_EH) has been digitally signed with external signatures using Entrust PKI.
Signatures manifested as of 11/5/2011 6:28:07 AM, signing status at this time: Completed (1 of 1 signatures)
Approved for report publication by Poirier Kevin in East Hanover at Sat, 05 Nov 2011 02:19:57 AM EDT

SOP	Standard Operating Procedure
TB	Total Bilirubin
TDF	Tenofovir Disiproxil Fumarate
UA	Urine Analysis(es)
ULN	Upper Limit of Normal
WBC	White Blood Cell Count
WHO	World Health Organization
WOCBP	Women of Child-Bearing Potential

This document (090095af830c511d in docbase CREDI_EH) has been digitally signed with external signatures using Entrust PKI.
Signatures manifested as of 11/5/2011 6:28:07 AM, signing status at this time: Completed (1 of 1 signatures)
Approved for report publication by Poirier Kevin in East Hanover at Sat, 05 Nov 2011 02:19:57 AM EDT

Glossary of terms

ALT flare	Severe ALT elevation with HBV viremia during continued or upon discontinuation of study treatment. ALT is persistently elevated to levels ≥ Baseline ALT level.
ALT level reduction	Reduction in ALT level from Baseline
Assessment	A procedure used to generate data required by the study
Baseline Visit	Day 1 of treatment for patient schedule of events from which all subsequent visits are determined.
Cockcroft-Gault method for estimated calculated creatinine clearance	(140-age) (ideal body weight in kg) / (72 X Cr_s) (Cr_s =serum creatinine mg/dL); for women multiply result by 0.85; note if Actual Body Weight (ABW) is lower than Ideal Body Weight (IBW), use ABW. IBW is calculated as follows: (males) IBW = 50 kg + 2.3 kg X inches>60 inches, (females) IBW = 45.5 kg + 2.3 kg X inches >60 inches
Control drug	A study drug used as a comparator to reduce assessment bias, preserve blinding of investigational drug, assess internal study validity, and/or evaluate comparative effects of the investigational drug
Early Discontinuation Visit	Day of final clinical and laboratory evaluation during the Treatment Phase if a patient has prematurely withdrawn from the study and includes all evaluations performed at Week 104 Visit
Enrollment	Point/time of patient entry into the study; the point at which informed consent must be obtained (i.e., prior to starting any of the procedures described in the protocol)
HBV DNA reduction	Change in $\log_{10}$ copies/mL of HBV DNA from Baseline
HBV DNA suppression	Serum HBV DNA < 3 $\log_{10}$ copies/mL on two successive visits in a patient with HBV DNA ≥ 5 $\log_{10}$ copies/mL (at least one million copies per mL) at baseline
Investigational drug	The study drug whose properties are being tested in the study; this definition is consistent with US CFR 21 Section 312.3 and is synonymous with “investigational new drug.”
Maintained ALT Normalization	ALT Normalization maintained to last treatment visit with no two intervening consecutive disqualifying values
Maintained HBV DNA Suppression	HBV DNA Suppression maintained to last treatment visit with no two intervening consecutive disqualifying values
Medication number	A unique identifier on the label of each medication package in studies that dispense medication using an IVR system
Patient number	A number assigned to each patient who enrolls in the study; when combined with the center number, a unique identifier for each patient in the study is created.
PCR	An acronym for Polymerase Chain Reaction. PCR is a technique which is used to amplify the number of copies of a specific region of DNA, in order to produce enough DNA to be adequately tested.
PCR negative	Serum HBV DNA will be considered to be non-detectable if it is below 300 copies/mL, the lower limit of quantification (LLOQ) of the COBAS Amplicor HBV Monitor™ assay used in this study.
Phase	A major subdivision of the study timeline; begins and ends with

This document (090095af830c511d in docbase CREDI_EH) has been digitally signed with external signatures using Entrust PKI.
Signatures manifested as of 11/5/2011 6:28:07 AM, signing status at this time: Completed (1 of 1 signatures)
Approved for report publication by Poirier Kevin in East Hanover at Sat, 05 Nov 2011 02:19:57 AM EDT

	major study milestones such as enrollment, completion of treatment, etc.
Period	A minor subdivision of the study timeline; divides phases into smaller functional segments such as screening, baseline, titration, washout, etc.
Post-treatment Follow-up phase	Phase of the study that is 16 weeks in duration starting after the 104-week Treatment Phase.
Premature patient withdrawal	Point/time when the patient exits from the study prior to the planned completion of all study drug administration and assessments; at this time all study drug administration is discontinued and no further assessments are planned
Roadmap concept	A schedule for monitoring and defining clinically meaningful on-treatment responses with the goal of enhancing patient disease management. The ‘roadmap concept’ applied in this study approach is an on-treatment monitoring and management strategy for patients receiving oral therapy and treatment adjustment based on the serum HBV DNA levels at 24 weeks (Keeffe et al 2007).
Study drug	Any drug administered to the patient as part of the required study procedures; includes investigational drug and any control drugs
Study drug discontinuation	Point/time when patient permanently stops taking study drug for any reason; may or may not also be the point/time of premature patient withdrawal
Treatment Phase visit	Visit that occurs during the phase of the protocol when patients receive study drug and have on therapy evaluations
Variable	Information used in the data analysis; derived directly or indirectly from data collected using specified assessments at specified timepoints
Virologic Breakthrough	For the subgroup of patients on treatment who achieve HBV DNA **≥** 1 $\log_{10}$ copies/mL reduction from baseline on two consecutive visits, Virologic Breakthrough is defined as HBV DNA ≥ 1 $\log_{10}$ copies/mL from nadir on two consecutive visits. Patients who have not achieved a reduction from baseline in HBV DNA but do exhibit an increase ≥ 1 $\log_{10}$ may not be indicative of viral breakthrough but could be a result of treatment failure instead**.**
Virologic response	HBeAg loss and HBV DNA < 3 $\log_{10}$ copies/mL

This document (090095af830c511d in docbase CREDI_EH) has been digitally signed with external signatures using Entrust PKI.
Signatures manifested as of 11/5/2011 6:28:07 AM, signing status at this time: Completed (1 of 1 signatures)
Approved for report publication by Poirier Kevin in East Hanover at Sat, 05 Nov 2011 02:19:57 AM EDT

Amendment 3

Amendment rationale

A critical assay that has been used throughout the entire study that measures HBV DNA reduction will no longer be available from the manufacturer. Other assay systems are available, but would not be a direct comparison against all previously collected data and would not be able to add any value to the safety or efficacy parameters of this study. The current assay system will only remain valid until December 31, This amendment will shorten the safety follow up period for patients whose final visit would occur in 2012. The lack of assay availability in 2012 does not impact the quality or validity of earlier assay data.

This change will reduce the follow up period for a select group of patients from 16 weeks to 12 weeks and will effect a maximum of 15 patients. The select group of patients correlates to those impacted by assay availability.

This reduction in follow up period will in no way impact the safety of the patients and will not effect any efficacy endpoints. Reduction in follow-up in the 15 patients is beyond the treatment period and does not impact patient safety, the integrity of safety assessments or efficacy endpoint at 104 weeks.

Current status is that all patients have now completed the treatment period and the remaining patients are all in the safety follow up period of the trial.

Changes to the protocol

This change will reduce the follow up period and change the completion criteria for a select group of patients from 16 weeks to 12 weeks and will effect a maximum of 15 patients.

6.5.13 Study completion and post-study treatment

A change in the completion guidelines for a select group of patients who would have their week 120 visit occur in the year 2012 is necessitated for the following reason: A critical assay that has been used throughout the entire study will no longer be available from the manufacturer. The current assay system will only remain valid until December 31, 2011. This unavailability of the HBV DNA assay will shorten the safety follow up period for patients whose final visit would occur in 2012.

Study completion for those patients who are effected by this unavailability of the HBV DNA assay will be defined as those patients who have met the screening requirements, attended the Baseline Day 1 visit, completed the 104 week Treatment Phase of the study and the shortened 12 week Follow-up Phase of the study.

This document (090095af830c511d in docbase CREDI_EH) has been digitally signed with external signatures using Entrust PKI.
Signatures manifested as of 11/5/2011 6:28:07 AM, signing status at this time: Completed (1 of 1 signatures)
Approved for report publication by Poirier Kevin in East Hanover at Sat, 05 Nov 2011 02:19:57 AM EDT

A copy of this amended protocol will be sent to the Institutional Review Board (IRBs)/Independent Ethics Committee (IECs) and Health Authorities. The changes described in this amended protocol are non-substantial and do not require IRB/IEC approval prior to implementation

Summary of previous amendments

Amendment 2: released March 3, 2009

Amendment 2 opened up the study to the total population of HBeAg positive CHB patients eligible for study and was consistent with international treatment guidelines (HBV DNA ≥ 5 log10 copies/ml (no upper limit) and ALT 1.3-10x ULN). This broader inclusion criteria was expected to result in higher rates of recruitment which ensured the successful completion of recruitment of all outstanding patients in the study. Patients with higher baseline HBV DNA levels are at higher risk of not achieving a PCR negative result at week 24 compared to those with lower HBV DNA baseline viral loads. Using the more open inclusion criteria, did result in greater numbers of patients with higher baseline HBV DNA being recruited, resulting in more patients requiring treatment intensification with tenofovir at week 24. This roadmap strategy maximizes the chances of achieving successful long term clinical outcomes (PCR negative results at week 104 and low risk of resistance) and is the central premise of the Roadmap Concept being prospectively studied in Study 2410. The statistical section of the Study has been updated to include an additional subgroup analysis prospectively exploring the effect of baseline characteristics on week 104 outcomes. In addition, “Exclusion criteria 8” has been revised in Amendment 2, to allow patients to have a 6 month washout period to IFN or other immodulatory treatment vs. the 12 month time period previously stipulated. As IFN therapy may achieve a sustained off therapy response in a proportion of patients, it should not make a difference whether the patient has a 6 or 12 month washout our period.

Amendment 2 has also clarified the potential treatment of patients during the “post treatment follow-up period”. Following the treatment period with the study drug(s), this period is usually aimed at collecting safety data. The investigators are asked to continue to evaluate the clinical results of the patient and if required, to provide additional HBV treatment to patients, according to their clinical judgment and practice. No further study drug will be provided by the sponsor during this 16 week post treatment follow-up period.

Amendment 1: released April 16, 2008

This amendment was designed to modify the inclusion/exclusion criteria in an effort to optimize responses to telbivudine treatment in patients with HBeAg-positive chronic hepatitis B (CHB). The GLOBE trial identified baseline patient characteristics, including ALT and HBV DNA levels, which were predictive of optimal treatment responses and were incorporated into the inclusion/exclusion criteria with this amendment. This amendment has also served to clearly align the visit assessment schedule (Table 7-1) and section 6.5.8 of the protocol as to which assessments was required in the management of patients discontinued from study prematurely. Also, a minor revision to the visit assessment schedule (Table 7-1)

This document (090095af830c511d in docbase CREDI_EH) has been digitally signed with external signatures using Entrust PKI.
Signatures manifested as of 11/5/2011 6:28:07 AM, signing status at this time: Completed (1 of 1 signatures)
Approved for report publication by Poirier Kevin in East Hanover at Sat, 05 Nov 2011 02:19:57 AM EDT

has reflected a change in timing for the HBV genotype assay. Patients have had the HBV genotype assay preformed at the Baseline Visit.

This document (090095af830c511d in docbase CREDI_EH) has been digitally signed with external signatures using Entrust PKI.
Signatures manifested as of 11/5/2011 6:28:07 AM, signing status at this time: Completed (1 of 1 signatures)
Approved for report publication by Poirier Kevin in East Hanover at Sat, 05 Nov 2011 02:19:57 AM EDT

Protocol synopsis

Title of study: A single-arm, multinational, two year study evaluating the efficacy and safety of lead-in telbivudine for 24 weeks with or without tenofovir treatment intensification in adult patients with HBeAg-positive chronic hepatitis B.

Purpose and rationale: The present study design is in keeping with the ***roadmap concept***, developed by HBV experts to optimize therapy in patients with chronic hepatitis B (CHB). Central to this concept is the use of on-treatment monitoring strategies of early virologic responses which may be predictive of improved outcomes, including reduced risk of antiviral resistance. Serum HBV DNA level at week 24 can be used to characterize virologic response as complete (< 300 copies/mL), partial (< 4 $\log_{10}$ copies/mL) and inadequate response (>4 $\log_{10}$ copies/mL). Strategies for managing patients in each of these categories were recommended and include the addition of a second antiviral agent (Keeffe et al 2007).

Utilizing the ***roadmap concept*** , this study will investigate the treatment approach strategy for CHB by evaluating the use of telbivudine (600 mg/day) treatment for patients with HBeAg-positive CHB with an option to intensify treatment at Week 24 by adding tenofovir disoproxil fumarate (300 mg/day) for patients who do not achieve HBV DNA non-detectability.

The key study design feature of treatment intensification with tenofovir among subjects whose HBV DNA level remains detectable after 24 weeks of treatment is based on data from the GLOBE phase III trial in adult patients with CHB which revealed that viral load after 24 weeks of telbivudine treatment was linked to efficacy outcomes after 1 and 2 years. A greater percentage of patients who were HBV DNA non-detectable at 24 weeks of therapy with telbivudine were HBV non-detectable, had undergone HBeAg seroconversion and ALT normalization and had significantly less resistance at 1 and 2 years of therapy, compared with those patients who were HBV DNA detectable at week 24 (DiBisceglie et al 2006; Marcellin et al 2007). Patients who remain detectable at week 24 will also receive tenofovir, possibly leading to improved anti-viral efficacy, clinical outcomes, and lower the risk of resistance at 2 years. Tenofovir has a non-overlapping resistance profile with telbivudine, and its addition at week 24 should improve antiviral efficacy outcomes and most likely result in a lower rate of resistance after two years of treatment in this subset of patients.

In summary, more effective treatment regimens are needed for patients with CHB. In particular, there is mounting interest in exploring the role of potent anti-viral combination therapies to further enhance longer term antiviral and clinical outcomes and reduce the rate of resistance development. This study evaluates the role of two potent antivirals, telbivudine and tenofovir, in combination regimen utilizing the roadmap concept of treatment intensification in patients who do not achieve a non-detectable HBV DNA by week 24 of treatment. The in vitro findings which suggest additive efficacy when using both drugs in combination is intriguing.

This document (090095af830c511d in docbase CREDI_EH) has been digitally signed with external signatures using Entrust PKI.
Signatures manifested as of 11/5/2011 6:28:07 AM, signing status at this time: Completed (1 of 1 signatures)
Approved for report publication by Poirier Kevin in East Hanover at Sat, 05 Nov 2011 02:19:57 AM EDT

Objectives: The primary objective of the study is to determine if telbivudine early non-responders can achieve an antiviral response with the addition of tenofovir.

The key secondary objectives of the study are:

1. To estimate the rate of virologic breakthrough up to week 48 and week 104
2. To assess the rate of treatment-emergent genotypically confirmed HBV resistance associated with viral breakthrough up to weeks 48 and 104

Other secondary objectives of the study include:

3. Assessment of HBV DNA non-detectability, reduction in HBV DNA from baseline and sustained reduction in HBV DNA over the course of study
4. Assessment of HBeAg loss and HBeAg seroconversion (defined as loss of HBeAg and development of HBeAb) over course of study
5. To describe the ALT normalization rate at weeks 52 and 104

Exploratory objectives of the study are:

To explore treatment outcomes at week 104 in the two subgroups of patients defined according to serum HBV DNA at week 24 – i) HBV DNA non-detectable without treatment intensification (telbivudine monotherapy) and ii) HBV DNA detectable with treatment intensification (add-on tenofovir)

Assessment of HBsAg loss and HBsAg seroconversion (defined as loss of HBsAg and development of HBsAb) over course of study

To assess the incidence and degree of elevations in CK levels and explore the relationship, if any, between CK elevations and musculoskeletal events

To assess safety (including ALT flares) in patients over course of study

Population: This is multinational study where the study population will be comprised of 100 outpatients with HBeAg-positive chronic hepatitis B.

Inclusion/Exclusion criteria:

Patients must meet all of the following inclusion criteria:

1. Male or female, at least 18 years of age.
2. Documented HBeAg positive CHB defined by all of the following:
   - Clinical history compatible with CHB
   - Detectable serum HBsAg at the Screening visit and at least 6 months prior
   - HBeAg positive at the Screening visit
   - HBeAb negative at the Screening visit
   - Serum HBV DNA level ≥ 5 $\log_{10}$ copies/mL as determined by the COBAS Amplicor HBV PCR assay (LLOD = 300 copies / mL) at the central study laboratory at Screening visit
   - Evidence of chronic liver inflammation, documented by previous history of elevated serum ALT and /or AST levels (at least two elevated ALT or AST

This document (090095af830c511d in docbase CREDI_EH) has been digitally signed with external signatures using Entrust PKI.
Signatures manifested as of 11/5/2011 6:28:07 AM, signing status at this time: Completed (1 of 1 signatures)
Approved for report publication by Poirier Kevin in East Hanover at Sat, 05 Nov 2011 02:19:57 AM EDT

values spanning six months or more, documented in available records) with or without prior liver biopsy that is consistent with CHB

- For patients with cirrhosis, clinical history compatible with compensated liver disease
- Elevated serum ALT level (1.3 – 10 x ULN) at the Screening visit

3. Patient is willing and able to comply with the study drug regimen and all other study requirements.
4. The patient is willing and able to provide written informed consent to participate in the study.

Patients will be excluded from the study for any of the following reasons:

1. History of hypersensitivity to any of the study drugs or to drugs with similar chemical structures.
2. Patient is pregnant or breastfeeding.
3. Women of child-bearing potential (WOCBP), defined as all women physiologically capable of becoming pregnant, including women whose career, lifestyle, or sexual orientation precludes intercourse with a male partner and women whose partners have been sterilized by vasectomy or other means. The exception of the aforementioned criteria will be given if they meet the following definition of post-menopausal: 12 months of natural (spontaneous) amenorrhea or 6 months of spontaneous amenorrhea with serum FSH levels >40 mIU/ml (IU/L) or 6 weeks post surgical bilateral oophorectomy with or without hysterectomy or have been surgically sterilized (e.g., bilateral tubal ligation) or the patient must agree to use two methods of birth control. This is any combination of hormonal contraception (implantable, patch, oral or injection), IUD, male or female condom with spermicidal gel, diaphragm, sponge or cervical cap. Women of childbearing potential must have a negative serum beta-human chorionic gonadotropin (β-HCG) during Screening.
4. Is a male who is capable of reproduction, defined as all men physiologically capable of producing sperm, including men whose career, lifestyle, or sexual orientation precludes intercourse with a female partner and men who have been sterilized (vasectomy) or whose partners have been sterilized by surgical bilateral oophorectomy with or without hysterectomy, bilateral tubal ligation or other means, UNLESS the female partner meets the following definition of post-menopausal: 12 months of natural (spontaneous) amenorrhea or 6 months of spontaneous amenorrhea with serum FSH levels >40 mIU/ml (IU/L) or the patient must agree to use two methods of birth control. This is any combination of hormonal contraception (implantable, patch ,oral or injection), IUD, male or female condom with spermicidal gel, diaphragm, sponge or cervical cap.
5. Patient is co-infected with HCV, HDV, or HIV.
6. Patients who have previously been involved in a trial with telbivudine.

This document (090095af830c511d in docbase CREDI_EH) has been digitally signed with external signatures using Entrust PKI.
Signatures manifested as of 11/5/2011 6:28:07 AM, signing status at this time: Completed (1 of 1 signatures)
Approved for report publication by Poirier Kevin in East Hanover at Sat, 05 Nov 2011 02:19:57 AM EDT

7. Patient has received nucleoside or nucleotide drugs whether approved or investigational at any time.
8. Patient has received IFN or other immunomodulatory treatment in the 6 months before Screening for this study.
9. Patient has a history of or clinical signs/symptoms of hepatic decompensation such as ascites, esophageal variceal bleeding, hepatic encephalopathy, hepatorenal syndrome, hepatic hydrothorax, hepatopulmonary syndrome or spontaneous bacterial peritonitis, among others.
10. Patient has a medical condition that requires prolonged or frequent use of systemic acyclovir or famciclovir.
11. Patient has a history of malignancy of any organ system, treated or untreated, within the past 5 years whether or not there is evidence of local recurrence or metastases, with the exception of localized basal cell carcinoma of the skin. Patients with previous findings suggestive of possible hepatocellular carcinoma (HCC), should have the disease ruled out prior to entrance into the study.
12. Patient has one or more additional known primary or secondary causes of liver disease other than CHB, including steatohepatitis and autoimmune hepatitis among other liver diseases. Gilbert's syndrome and Dubin-Johnson syndrome are not considered exclusion criteria for this study.
13. History of any other acute or chronic medical condition that in the opinion of the investigator would make the patient unsuitable for inclusion into the study.
14. Patient is currently abusing alcohol or illicit drugs, or has a history of alcohol abuse or illicit substance abuse within the preceding two years. Please refer to Appendix 2.
15. Patient has a medical condition that requires frequent or prolonged use of systemic corticosteroids, although inhaled corticosteroids are allowed.
16. Patient has a history of clinical and laboratory evidence of chronic renal insufficiency.
17. Patient has a medical condition requiring the chronic or prolonged use of potentially hepatotoxic drugs or nephrotoxic drugs.
18. Patient has any other concurrent medical or social condition likely to preclude compliance with the schedule of evaluations in the protocol, or likely to confound the efficacy or safety observations of the study.
19. Use of other investigational drugs at the time of enrollment, or within 30 days or 5 half-lives of enrollment, whichever is longer.
20. Patient has a history of myopathy, myositis, or persistent muscle weakness.
21. Patient has any of the following laboratory values during Screening:

- Hemoglobin <11 g/dL (110 g/L) for men or <10 g/dL (100 g/L) for women
- Total WBC <3,500/mm^3 (3.5 x 10^9/Liter)
- Absolute neutrophil count (ANC) <1,500/mm^3 (1.5 x 10^9/Liter)
- Platelet count <75,000/mm^3 (75 x 10^9/Liter)

This document (090095af830c511d in docbase CREDI_EH) has been digitally signed with external signatures using Entrust PKI.
Signatures manifested as of 11/5/2011 6:28:07 AM, signing status at this time: Completed (1 of 1 signatures)
Approved for report publication by Poirier Kevin in East Hanover at Sat, 05 Nov 2011 02:19:57 AM EDT

- Serum amylases or lipase ≥ 1.5 x ULN
- Serum albumin <3.3 g/dL (33g/L)
- Total bilirubin ≥ 2.0 mg/dL (34.2 μmol/L)
- Estimated calculated serum creatinine clearance < 50 mL/min (0.48 ml/s) using the Cockcroft-Gault method with lean or ideal body weight; see Glossary of Terms (Cockcroft 1976)
- AFP > 50 ng/mL or μg/L (requires further work up per local medical standards)

Investigational and reference therapy:

- LDT 600 (telbivudine, Sebivo®) 600 mg, film-coated tablets
- Tenofovir disoproxil fumarate (Viread®) 300mg tablets

Study design: This is a prospective, single-arm, 104 week, multicenter, multinational, phase IV study to characterize the efficacy and safety of treatment with telbivudine with or without treatment intensification by adding tenofovir disoproxil fumarate at Week 24 in adult patients with HBeAg-positive chronic hepatitis B.

Sample size is 100 eligible patients who will be assigned to the following treatment arm as seen in Figure 4-1.

Telbivudine 600 mg tablet PO once daily for 104 weeks. Patients with HBV DNA ≥ 300 copies/ml at week 24 will initiate treatment intensification by adding tenofovir disoproxil fumarate 300 mg tablet PO once daily for the remaining 80 weeks of treatment.

Eligible patients will receive the first dose of study medication at the study Baseline Visit (Day 1). Subsequently, patients will return to the clinic at post-baseline Weeks 2, 4, 8, 12, 16, 24, 26, 30, 40, 48, 52, 60, 68, 76, 86, 96, and 104 with Post-treatment Follow-up visits at Weeks 108, 112, 116 and 120 (A change in the completion guidelines for a select group of patients who would have their week 120 visit occur in the year 2012 is necessitated for the following reason: A critical assay that has been used throughout the entire study will no longer be available from the manufacturer. The current assay system will only remain valid until December 31, 2011. This unavailability of the HBV DNA assay will shorten the safety follow up period for patients whose final visit would occur in 2012. This change will reduce the follow up period for a select group of patients from 16 weeks to 12 weeks and will effect a maximum of 15 patients. The select group of patients correlates to those impacted by assay availability.)

. At each of these visits, routine vital signs, clinical laboratory tests, adverse event inquiry, and other assessments will be performed.

Efficacy assessments:

Serum HBV DNA assessments

Serum HBV DNA determinations will be performed at a central reference laboratory through use of the COBAS Amplicor HBV Monitor assay (Roche Molecular

This document (090095af830c511d in docbase CREDI_EH) has been digitally signed with external signatures using Entrust PKI.
Signatures manifested as of 11/5/2011 6:28:07 AM, signing status at this time: Completed (1 of 1 signatures)
Approved for report publication by Poirier Kevin in East Hanover at Sat, 05 Nov 2011 02:19:57 AM EDT

Systems, Branchburg, NJ, USA), which utilizes polymerase chain reaction (PCR) methods and semi-automated sample readout technologies (threshold for detection 300 copies/mL). The COBAS Amplicor HBV Monitor assay has been used in the previously conducted Phase II and Phase III telbivudine studies, and thus results from this study will be able to be linked to existing data on telbivudine.

Serum samples for HBV DNA will be obtained during Screening to determine eligibility for the study. The Screening serum HBV DNA values must be ‡ 5 $\log_{10}$ copies/mL by the COBAS Amplicor HBV Monitor assay at the central study laboratory.

Serum samples for HBV DNA will be obtained during Screening and at the Baseline Visit (Day 1), and at every protocol-stipulated Treatment Phase visit (Weeks 2, 4, 8, 12, 16, 24, 30, 40, 48, 52, 60, 68, 76, 86, 96, and 104 or at the Early Discontinuation visit) and at all Post-treatment Follow-up visits.

Serologic markers

Other HBV serologic markers (HBsAg/Ab, HBeAg/Ab) will be assessed at the central study laboratory using standard commercially-available enzyme immunoassays. Serum for HBsAg/Ab and HBeAg/Ab will be collected during Screening and at the Baseline Visit (Day 1), and at every protocol-stipulated Treatment Phase visit (Weeks 52 and 104 or at the Early Discontinuation visit) and at the Week 120(A change in the completion guidelines for a select group of patients who would have their week 120 visit occur in the year 2012 is necessitated for the following reason: A critical assay that has been used throughout the entire study will no longer be available from the manufacturer. The current assay system will only remain valid until December 31, 2011. This unavailability of the HBV DNA assay will shorten the safety follow up period for patients whose final visit would occur in 2012. This change will reduce the follow up period for a select group of patients from 16 weeks to 12 weeks and will effect a maximum of 15 patients. The select group of patients correlates to those impacted by assay availability.)Post-treatment Follow-up visit.

ALT

Serum ALT normalization is an important clinically-relevant efficacy endpoint. Elevated serum ALT levels are thought to reflect underlying hepatitis disease activity (i.e., active liver inflammation). Correspondingly, ALT normalization is an accepted therapeutic goal in hepatitis studies as it is thought to reflect a substantial reduction in hepatic disease activity. ALT levels will be determined from serum samples obtained at all study visits, via standard central laboratory testing.

Other assessments:

Virologic Breakthrough and Resistance

Virologic breakthrough and treatment emergent HBV resistance that are associated with viral breakthrough, for patients treated with telbivudine with add-on therapy, and telbivudine monotherapy up to weeks 48 and 104 will be assessed. Virologic breakthrough will be defined as an increase of serum HBV DNA greater than 1 $\log_{10}$ copies/mL from nadir. The emergence of treatment-associated genotypic HBV

This document (090095af830c511d in docbase CREDI_EH) has been digitally signed with external signatures using Entrust PKI.
Signatures manifested as of 11/5/2011 6:28:07 AM, signing status at this time: Completed (1 of 1 signatures)
Approved for report publication by Poirier Kevin in East Hanover at Sat, 05 Nov 2011 02:19:57 AM EDT

resistance will be investigated in all samples with virologic breakthrough and the results expressed as cumulative rates of resistance by treatment week 48 and week 104.

Assessment of adverse events.

Physical examinations, vital signs, height (during Screening only), weight, and pregnancy tests.

Routine laboratory tests including biochemistry, prothrombin time, urinalysis.

Data analysis: To evaluate the primary objective, the proportion of patients (response rate) who achieve HBV DNA non-detectability at week 52 will be estimated along with a 95% confidence interval.

The rate of treatment-emergent viral breakthrough and genotypically confirmed HBV resistance associated with these viral breakthrough will be similarly assessed up to weeks 48 and 104. The HBV DNA level,, HBV DNA reduction from baseline, HBV DNA non-detectability rate and ALT normalization rate will be summarized and plotted over time with 95% confidence intervals.

This document (090095af830c511d in docbase CREDI_EH) has been digitally signed with external signatures using Entrust PKI.
Signatures manifested as of 11/5/2011 6:28:07 AM, signing status at this time: Completed (1 of 1 signatures)
Approved for report publication by Poirier Kevin in East Hanover at Sat, 05 Nov 2011 02:19:57 AM EDT

1 Background

1.1 Chronic Hepatitis B: Disease overview

Chronic hepatitis B (CHB) is a common global health problem (Keeffe et al 2006), with approximately 33% of people worldwide (about 2 billion people) who have been infected with HBV at some time in their lives with 50 million new cases diagnosed annually (Merican et al 2000). After acute HBV infection, 3-5% of adults and up to 95% of children go on to develop CHB (Hoofnagle et al 2007). Approximately 5% of the world's population (350-400 million people) are chronically HBV-infected. HBV infection may cause up to 80% of primary hepatic carcinomas; and about 25% of chronic HBV carriers (over 1 million people annually) eventually die from HBV-related end stage complications such as cirrhosis-related liver failure and hepatocellular carcinoma.

1.2 Current treatments for CHB

The goal of therapy of CHB is suppression of HBV replication in an effort to prevent the development of hepatocellular carcinoma (HCC) and progression to cirrhosis and complications thereof including portal hypertension, ascites formation, and variceal bleeding, among others. Reduction in viral load has been shown to correlate with reduction in the incidence of long term events, such as hepatic decompensation and HCC (Liaw 2005). Recent reports have shown that maximal HBV DNA reduction early in treatment has been linked to better long-term clinical outcomes (Lai et al 2005b, Chen et al 2006). Thus, there is an increasing rationale for maximizing viral suppression early in treatment and maintaining it as low as possible in order to improve long-term virologic and clinical outcomes in HBeAg-positive patients.

Six therapies are available globally for the treatment of CHB including parenterally administered interferon or peginterferon alpha-2a (Pegasys®) (Roche U.S. product label, 2005), oral nucleoside analogues lamivudine (LAM), entecavir (ETV), telbivudine (LDT) and the oral nucleotide analogue adefovir dipivoxil (ADV).

The main attribute of immunomodulators, such as interferons, is the induction of is the seroconversion (Janssen et al 2005). Limitations to treatment with immunomodulators include significant side effects such as flu-like symptoms, depression and hematologic toxicity among others, which results in poor treatment tolerability and premature discontinuation of therapy (Perrillo et al 1990; Wong et al 1993; Hoofnagle and Di Bisceglie 1997; Lee 1997; Renault and Hoofnagle 1989; Perrillo 1993; Intron®-A product label 2002). Furthermore, treatment is costly and contraindicated in patients with decompensated liver disease and two thirds of patients do not respond to immunomodulators.

The strength of the nucleos(t)ide therapy is in the potent reduction of HBV DNA by directly inhibiting the HBV polymerase. Given their ease of administration, side effect profile and ability to be given for long periods of time, antivirals are an attractive alternative to immunomodulators for treatment of CHB. Limitations of antiviral therapy include modest antiviral efficacy (LAM and ADV), frequent development of resistance (LAM), potential

This document (090095af830c511d in docbase CREDI_EH) has been digitally signed with external signatures using Entrust PKI.
Signatures manifested as of 11/5/2011 6:28:07 AM, signing status at this time: Completed (1 of 1 signatures)
Approved for report publication by Poirier Kevin in East Hanover at Sat, 05 Nov 2011 02:19:57 AM EDT

nephrotoxicity (ADV) and carcinogenicity and teratogenicity issues (ETV) (Epivir-HBV® product label, 2004; Hepsera® product label, 2004; Baraclude™ product label, 2005).

1.2.1 Combination Therapy with Antivirals

Combination drug regimens of a nucleotide and nucleoside was explored by a panel of experts at a conference organized by the NIH in April 2006 on the management of the hepatitis B virus. Combination therapy with 2 antivirals, specifically a nucleoside and nucleotide combination, theoretically would have greater antiviral efficacy than either drug used alone and probably a lower rate of anti-viral resistance (Lok et al 2007).

Three published studies to date have evaluated combinations of antiviral therapy in the treatment of CHB. One of these studies compared lamivudine in combination with adefovir versus lamivudine alone in HBeAg-positive patients. Antiviral response did not differ between the two groups at weeks 52 and 104, although there was less resistance in the combination arm (15%) when compared to the lamivudine monotherapy arm (43%) (Sung et al 2003). Another study assessing the antiviral effects of telbivudine in combination with lamivudine showed similar results to those obtained with telbivudine monotherapy alone (Lai et al 2005).

1.2.2 Telbivudine

Telbivudine (β-L-2'-deoxythymidine) is a highly specific and potent inhibitor of HBV replication in vitro (Bryant et al 2001). In the GLOBE trial, a phase III randomized comparison of telbivudine versus LAM, telbivudine demonstrated superiority over LAM in HBeAg-positive and HBeAg negative patients on most key study endpoints after 52 and 104 weeks, respectively.

Clinical studies conducted so far have demonstrated that telbivudine is safe and well-tolerated. The major safety finding observed in the clinical studies was an increase in serum levels of creatine kinase (CK) which were more common among telbivudine than lamivudine treated patients. Treatment was rarely interrupted or discontinued as a result of elevated CK. There were rare (<1%) reports of myopathy among telbivudine treated patients in the GLOBE study, all of which resolved. Telbivudine has so far been approved for the treatment of CHB in many parts of the world, including the United States, EU, China, Korea, and Taiwan among others.

1.2.3 Tenofovir

Tenofovir disoproxil fumarate, a nucleotide analogue, is a potent antiviral agent with activity against both HIV and HBV, and whose HBV antiviral efficacy was shown in several clinical trials (Ristig et al 2002, van Bömmel et al 2004, van Bömmel et al 2006). Results from a Phase III trial comparing tenofovir to adefovir treatment in 266 patients with HBeAg- positive CHB demonstrated that significantly greater tenofovir- than adefovir-treated patients (67% versus 12%, p<0.001) achieved the primary composite endpoint (HBV DNA <400 copies/ml and histologic improvement: > 2-point reduction in the Knodell necroinflammatory score without worsening in fibrosis). In addition, significantly more tenofovir- than adefovir-treated patients (69% versus 9%, p<0.001) had HBV DNA < 169 copies/ml as measured by the

This document (090095af830c511d in docbase CREDI_EH) has been digitally signed with external signatures using Entrust PKI.
Signatures manifested as of 11/5/2011 6:28:07 AM, signing status at this time: Completed (1 of 1 signatures)
Approved for report publication by Poirier Kevin in East Hanover at Sat, 05 Nov 2011 02:19:57 AM EDT

Roche COBAS Taqman assay (Heathcote et al 2007). Tenofovir was well tolerated in the study and there were no discontinuations of the drug due to an adverse event.

1.3 Drug-Drug Interaction Studies

In vitro combination studies conducted with telbivudine and tenofovir demonstrated additive to weakly synergistic anti-HBV activity compared to antiviral activity of treatments with telbivudine or tenofovir alone. The enhanced efficacy was additive and there was no evidence of cytotoxicity (Patti et al 2007).

A Phase I study was conducted to assess the potential drug-drug interactions between telbivudine (600 mg/day) and tenofovir disoproxil fumarate (300mg/day) (NV-02B-028) in healthy subjects.

Plasma pharmacokinetic (PK) parameters of telbivudine at steady-state were similar when telbivudine was administered alone and in combination with tenofovir. Similarly, steady-state PK parameters of tenofovir were also comparable when tenofovir was administered alone and in combination with telbivudine. Study drugs were in general well tolerated when administered in the combination. Results from this study indicated that there was no appreciable plasma PK drug-drug interaction between telbivudine and tenofovir in healthy human subjects. No serious adverse events were reported in this study and no subjects were discontinued as a result of adverse events.

2 Purpose and rationale

The present study design is in keeping with the ***roadmap concept***, developed by HBV experts to optimize therapy in patients with CHB. Central to this concept is the use of on-treatment monitoring strategies of early virologic responses which may be predictive of improved outcomes, including reduced risk of antiviral resistance. Serum HBV DNA level at week 24 can be used to characterize virologic response as complete (< 300 copies/mL), partial (< 4 log_{10} copies/mL) and inadequate response (>4 log_{10} copies/mL). Strategies for managing patients in each of these categories were recommended and include the addition of a second antiviral agent (Keeffe et al 2007).

Utilizing the ***roadmap concept*** , this study will investigate the treatment approach strategy for chronic hepatitis B (CHB) by evaluating the use of telbivudine (600 mg/day) treatment for patients with HBeAg-positive CHB with an option to intensify treatment at Week 24 by adding tenofovir disoproxil fumarate (300 mg/day) for patients who do not achieve HBV DNA non-detectability.

The key study design feature of treatment intensification with tenofovir among subjects whose HBV DNA level remains detectable after 24 weeks of treatment is based on data from the GLOBE phase III trial in adult patients with CHB which revealed that viral load after 24 weeks of telbivudine treatment was linked to efficacy outcomes after 1 and 2 years. A greater percentage of patients who were HBV DNA non-detectable at 24 weeks of therapy with telbivudine were HBV non-detectable, had undergone HBeAg seroconversion and ALT normalization and had significantly less resistance at 1 and 2 years of therapy, compared with those patients who were HBV DNA detectable at week 24 (DiBisceglie et al 2006, Marcellin et al 2007). Patients who remain detectable at week 24 will also receive tenofovir, possibly

This document (090095af830c511d in docbase CREDI_EH) has been digitally signed with external signatures using Entrust PKI.
Signatures manifested as of 11/5/2011 6:28:07 AM, signing status at this time: Completed (1 of 1 signatures)
Approved for report publication by Poirier Kevin in East Hanover at Sat, 05 Nov 2011 02:19:57 AM EDT

leading to improved anti-viral efficacy, clinical outcomes, and lower the risk of resistance at 2 years. Tenofovir has a non-overlapping resistance profile with telbivudine, and its addition at week 24 should improve antiviral efficacy outcomes and most likely result in a lower rate of resistance after two years of treatment in this subset of patients.

In summary, more effective treatment regimens are needed for patients with CHB. In particular, there is mounting interest in exploring the role of potent anti-viral combination therapies to further enhance longer term antiviral and clinical outcomes and reduce the rate of resistance development. This study evaluates the role of two potent antivirals, telbivudine and tenofovir, in combination regimen utilizing the roadmap concept of treatment intensification in patients who do not achieve a non-detectable HBV DNA by week 24 of treatment. The in vitro findings which suggest additive efficacy when using both drugs in combination is intriguing.

3 Objectives

3.1 Primary objective

The primary objective of the study is to determine if telbivudine early non-responders can achieve an antiviral response with the addition of tenofovir.

3.2 Secondary objectives

The key secondary objectives of the study are:

1. To estimate the rate of virologic breakthrough up to week 48 and week 104
2. To assess the rate of treatment-emergent genotypically confirmed HBV resistance associated with viral breakthrough up to weeks 48 and 104

Other secondary objectives of the study include:

3. Assessment of HBV DNA non-detectability, reduction in HBV DNA from baseline and sustained reduction in HBV DNA over the course of study
4. Assessment of HBeAg loss and HBeAg seroconversion (defined as loss of HBeAg and development of HBeAb) over course of study
5. To describe the ALT normalization rate at weeks 52 and 104

3.3 Exploratory objectives

1. To explore treatment outcomes at week 104 in the two subgroups of patients defined according to serum HBV DNA at week 24 – i) HBV DNA non-detectable without treatment intensification (telbivudine monotherapy) and ii) HBV DNA detectable with treatment intensification (add-on tenofovir)
2. Assessment of HBsAg loss and HBsAg seroconversion (defined as loss of HBsAg and development of HBsAb) over course of study
3. To assess the incidence and degree of elevations in CK levels and explore the relationship, if any, between CK elevations and musculoskeletal events
4. To assess safety (including ALT flares) in patients over course of study

This document (090095af830c511d in docbase CREDI_EH) has been digitally signed with external signatures using Entrust PKI.
Signatures manifested as of 11/5/2011 6:28:07 AM, signing status at this time: Completed (1 of 1 signatures)
Approved for report publication by Poirier Kevin in East Hanover at Sat, 05 Nov 2011 02:19:57 AM EDT

4 Study design

This is a prospective, single-arm, 104 week, multicenter, multinational, phase IV study to characterize the efficacy and safety of treatment with telbivudine with or without treatment intensification by adding tenofovir disoproxil fumarate at Week 24 in adult patients with HBeAg-positive chronic hepatitis B.

The treatment group is illustrated in Figure 4-1.

Figure 4-1 Study design

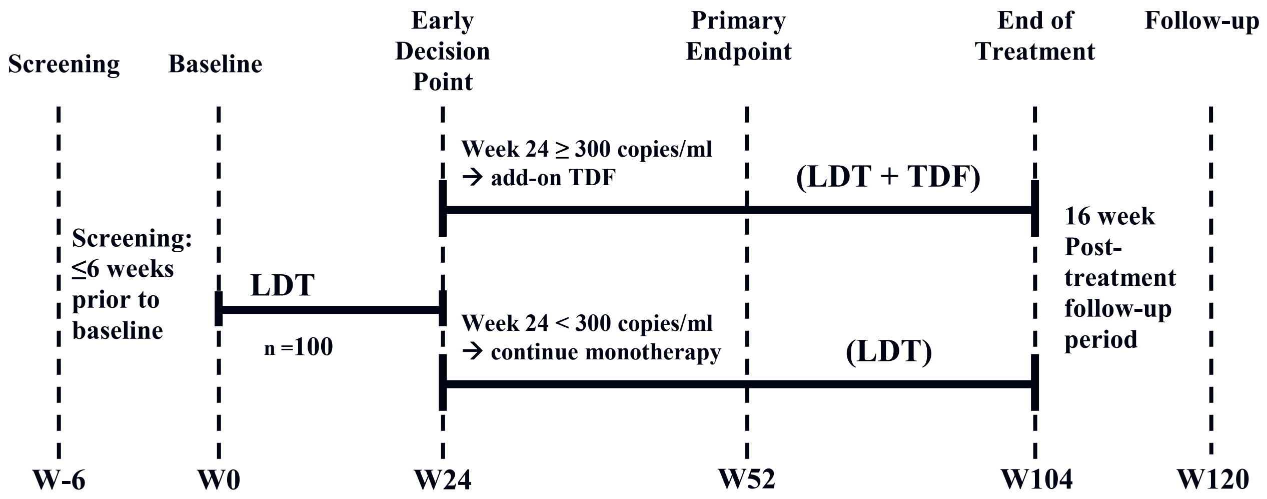

The study consists of 4 periods: Screening (≤ 6 weeks prior to Baseline Visit), Baseline Visit (Day 1), Treatment Phase (104 weeks) and Post-treatment Follow-up (16 weeks). Patient eligibility will be established during the Screening period. In the instance a screened patient fails to meet entry criteria based on laboratory outcomes, the patient can be retested one additional time for the analyte(s) previously failed in order to reassess eligibility. If the patient fails the retest, the patient should not be further evaluated, and dropped as a screen failure. Once eligible, patients will begin treatment with their first dose of study medication at the Baseline Visit (Day 1). Finally patients will be evaluated for safety and sustained treatment effects in the Post-treatment Follow-up phase. Data analyses are planned at two points during this study. The first analysis will be at 52 weeks (i.e., the primary efficacy analysis), and second consolidated analysis will be at the completion of the treatment phase at week 104 and the follow-up at week 120 (A change in the completion guidelines for a select group of patients who would have their week 120 visit occur in the year 2012 is necessitated for the following reason: A critical assay that has been used throughout the entire study will no longer be available from the manufacturer. The current assay system will only remain valid until December 31, 2011. This unavailability of the HBV DNA assay will shorten the safety follow up period for patients whose final visit would occur in 2012. This change will reduce the follow up period for a select group of patients from 16 weeks to 12 weeks and will effect a maximum of 15 patients. The select group of patients correlates to those impacted by assay availability.).

This document (090095af830c511d in docbase CREDI_EH) has been digitally signed with external signatures using Entrust PKI.
Signatures manifested as of 11/5/2011 6:28:07 AM, signing status at this time: Completed (1 of 1 signatures)
Approved for report publication by Poirier Kevin in East Hanover at Sat, 05 Nov 2011 02:19:57 AM EDT

5 Population

This is a multinational study where the study population will be comprised of 100 outpatients with HBeAg-positive chronic hepatitis B.

5.1 Inclusion criteria

Patients eligible for inclusion in this study have to fulfill all of the following criteria:

1. Male or female, at least 18 years of age.
2. Documented HBeAg positive CHB defined by all of the following:
    - Clinical history compatible with CHB
    - Detectable serum HBsAg at the Screening visit and at least 6 months prior
    - HBeAg positive at the Screening visit
    - HBeAb negative at the Screening visit
    - Serum HBV DNA level ≥ 5 $\log_{10}$ copies/mL, as determined by the COBAS Amplicor HBV PCR assay (LLOD = 300 copies / mL) at the central study laboratory at Screening visit
    - Evidence of chronic liver inflammation, documented by previous history of elevated serum ALT and /or AST levels (at least two elevated ALT or AST values spanning six months or more, documented in available records) with or without prior liver biopsy that is consistent with CHB
    - For patients with cirrhosis, clinical history compatible with compensated liver disease
    - Elevated serum ALT level (1.3 – 10 x ULN) at the Screening visit
3. Patient is willing and able to comply with the study drug regimen and all other study requirements.
4. The patient is willing and able to provide written informed consent to participate in the study.

5.2 Exclusion criteria

Patients will be excluded from the study for any of the following:

1. History of hypersensitivity to any of the study drugs or to drugs with similar chemical structures.
2. Patient is pregnant or breastfeeding.
3. Women of child-bearing potential (WOCBP), defined as all women physiologically capable of becoming pregnant, including women whose career, lifestyle, or sexual orientation precludes intercourse with a male partner and women whose partners have been sterilized by vasectomy or other means. The exception of the aforementioned criteria will be given if they meet the following definition of post-menopausal: 12 months of natural (spontaneous) amenorrhea or 6 months of spontaneous amenorrhea with serum FSH levels >40 mIU/ml (IU/L) or 6 weeks post surgical bilateral oophorectomy with or without hysterectomy or have been surgically sterilized (e.g., bilateral tubal ligation) or the patient must agree to use two methods of birth control. This is any combination of

This document (090095af830c511d in docbase CREDI_EH) has been digitally signed with external signatures using Entrust PKI.
Signatures manifested as of 11/5/2011 6:28:07 AM, signing status at this time: Completed (1 of 1 signatures)
Approved for report publication by Poirier Kevin in East Hanover at Sat, 05 Nov 2011 02:19:57 AM EDT

hormonal contraception (implantable, patch, oral or injection), IUD, male or female condom with spermicidal gel, diaphragm, sponge or cervical cap. Women of childbearing potential must have a negative serum beta-human chorionic gonadotropin (β-HCG) during Screening.

4. Is a male who is capable of reproduction, defined as all men physiologically capable of producing sperm, including men whose career, lifestyle, or sexual orientation precludes intercourse with a female partner and men who have been sterilized (vasectomy) or whose partners have been sterilized by surgical bilateral oophorectomy with or without hysterectomy, bilateral tubal ligation or other means, UNLESS the female partner meets the following definition of post-menopausal: 12 months of natural (spontaneous) amenorrhea or 6 months of spontaneous amenorrhea with serum FSH levels >40 mIU/ml (IU/L) or the patient must agree to use two methods of birth control. This is any combination of hormonal contraception (implantable, patch, oral or injection), IUD, male or female condom with spermicidal gel, diaphragm, sponge or cervical cap.
5. Patient is co-infected with HCV, HDV, or HIV.
6. Patients who have previously been involved in a trial with telbivudine.
7. Patient has received nucleoside or nucleotide drugs whether approved or investigational at any time. Please refer to Appendix 2.
8. Patient has received IFN or other immunomodulatory treatment in the 6 months before Screening for this study. Please refer to Appendix 2.
9. Patient has a history of or clinical signs/symptoms of hepatic decompensation such as ascites, esophageal variceal bleeding, hepatic encephalopathy, hepatorenal syndrome, hepatic hydrothorax, hepatopulmonary syndrome or spontaneous bacterial peritonitis.
10. Patient has a medical condition that requires prolonged or frequent use of systemic acyclovir or famciclovir. Please refer to Appendix 2.
11. History of malignancy of any organ system, treated or untreated, within the past 5 years whether or not there is evidence of local recurrence or metastases, with the exception of localized basal cell carcinoma of the skin. Patients with previous findings suggestive of possible HCC, should have the disease ruled out prior to entrance into the study.
12. Patient has one or more additional known primary or secondary causes of liver disease, other than CHB, including steatohepatitis and autoimmune hepatitis among other liver diseases. Note: Gilbert's syndrome and Dubin-Johnson syndrome are not considered exclusion criteria for this study.
13. History of any other acute or chronic medical condition that in the opinion of the investigator would make the patient unsuitable for inclusion into the study.
14. Patient is currently abusing alcohol or illicit drugs, or has a history of alcohol abuse or illicit substance abuse within the preceding two years. Please refer to Appendix 2.
15. Patient has a medical condition that requires frequent or prolonged use of systemic corticosteroids, although topical and inhaled corticosteroids are allowed.
16. Patient has a history of clinical and laboratory evidence of chronic renal insufficiency defined as an estimated serum creatinine clearance < 50 mL/min using the Cockcroft-Gault method.

This document (090095af830c511d in docbase CREDI_EH) has been digitally signed with external signatures using Entrust PKI.
Signatures manifested as of 11/5/2011 6:28:07 AM, signing status at this time: Completed (1 of 1 signatures)
Approved for report publication by Poirier Kevin in East Hanover at Sat, 05 Nov 2011 02:19:57 AM EDT

17. Patient has a medical condition requiring the chronic or prolonged use of potentially hepatotoxic drugs or nephrotoxic drugs. Please refer to the Appendix 2 list of prohibited concurrent medications.
18. Patient has any other concurrent medical or social condition likely to preclude compliance with the schedule of evaluations in the protocol, or likely to confound the efficacy or safety observations of the study.
19. Use of other investigational drugs at the time of enrollment, or within 30 days or 5 half-lives of enrollment, whichever is longer.
20. Patient has a history of myopathy, myositis, or persistent muscle weakness.
21. Patient has any of the following laboratory values during Screening:
    - Hemoglobin <11 g/dL (110 g/L) for men or <10 g/dL (100 g/L) for women
    - Total WBC <3,500/mm^3 (3.5 x 10^9/Liter)
    - Absolute neutrophil count (ANC) <1,500/mm^3 (1.5 x 10^9/Liter)
    - Platelet count <75,000/mm^3 (75 x 10^9/Liter)
    - Serum amylases or lipase ≥ 1.5 x ULN
    - Serum albumin <3.3 g/dL (33g/L)
    - Total bilirubin ≥ 2.0 mg/dL (34.2 µmol/L)
    - Estimated calculated serum creatinine clearance < 50 mL/min (0.48 ml/s) using the Cockcroft-Gault method with lean or ideal body weight; see Glossary of Terms (Cockcroft 1976)
    - AFP > 50 ng/mL or µg/L (requires further work up per local medical standards)

6 Treatment

6.1 Investigational and control drugs

The following drugs will be supplied:

- LDT 600 (telbivudine, Sebivo®) 600 mg, film-coated tablets
- Tenofovir disoproxil fumarate (Viread®) 300mg tablets

Telbivudine (Sebivo®) 600 mg tablets and Tenofovir disoproxil fumarate (Viread®) 300mg tablets will be supplied locally in commercial packs.

6.2 Treatment arm

Patients will be assigned to a single treatment arm (see Table 6-1).

Telbivudine 600 mg tablet PO once daily for 104 weeks. Patients with HBV DNA ≥ 300 copies/ml at week 24 will initiate add-on therapy of tenofovir disoproxil fumarate 300mg tablets PO once daily for the remaining 80 weeks of treatment. The investigator will initiate tenofovir add-on therapy within two weeks of central laboratory confirmation. Patients with with < 300 copies/ml at week 24 will continue to receive telbivudine monotherapy.

This document (090095af830c511d in docbase CREDI_EH) has been digitally signed with external signatures using Entrust PKI.
Signatures manifested as of 11/5/2011 6:28:07 AM, signing status at this time: Completed (1 of 1 signatures)
Approved for report publication by Poirier Kevin in East Hanover at Sat, 05 Nov 2011 02:19:57 AM EDT

Table 6-1 Treatment group assignment and instructions for study drug use

Treatment group	# Oral dose tablets	HBV DNA assessment at week 24	# Oral dose tablets	Treatment duration is for a total of 104 weeks
All patients begin with telbivudine monotherapy	1 x 600 mg telbivudine daily for first 24 weeks	HBV DNA < 300 copies/ml	1 x 600 mg telbivudine daily	Continue monotherapy x 80 remaining weeks [a]
		HBV DNA ≥ 300 copies/mL	1 x 600 mg telbivudine daily plus 1 x 300 mg tenofovir daily	Add-on and continue daily x 80 remaining weeks [a]

[a] medication should be taken in the morning at approximately the same time each day

6.3 Treatment assignment

At the Baseline Visit all patients who fulfill all the inclusion/exclusion criteria will be given the lowest available patient number. This number assigns them to the treatment arm and the investigator will enter the patient number in the eCRF.

6.4 Treatment blinding

This study will not have any treatment blinding.

6.5 Treating the patient

6.5.1 Patient numbering

Each patient is uniquely identified in the study by a combination of his/her center number and patient number. The center number is assigned by Novartis to the investigative site. Upon signing the informed consent form, the patient is assigned a patient number by the investigator. At each site the first patient is assigned patient number 1, and subsequent patients are assigned consecutive numbers (e.g., the second patient is assigned patient number 2, the third patient is assigned patient number 3). For studies using eCRFs, only the assigned patient number should be entered in the field labeled “Subject ID” on the EDC data entry screen. Once assigned to a patient, a patient number will not be reused. If the patient fails to be assigned to treatment for any reason, the reason for not being assigned to treatment will be entered on the Screening Log, and the Demography CRF/eCRF should also be completed.

6.5.2 Dispensing the study drug

Telbivudine 600 mg tablets and tenofovir disoproxil fumarate 300mg tablets will be supplied locally.

6.5.3 Study drug supply, storage and tracking

Telbivudine (Sebivo®) and tenofovir disoproxil fumarate (Viread®) will be supplied locally by the sponsor in commercial packages.

Study drugs must be received by a designated person at the study site, handled and stored safely and properly, and kept in a secured location to which only the investigator and

This document (090095af830c511d in docbase CREDI_EH) has been digitally signed with external signatures using Entrust PKI.
Signatures manifested as of 11/5/2011 6:28:07 AM, signing status at this time: Completed (1 of 1 signatures)
Approved for report publication by Poirier Kevin in East Hanover at Sat, 05 Nov 2011 02:19:57 AM EDT

designated assistants have access. Upon receipt, all study drugs should be stored according to the instructions specified on the drug labels. Clinical supplies are to be dispensed only in accordance with the protocol.

Medication labels will be in the local language and comply with the legal requirements of each country. They will include storage conditions for the drug, but no information about the patient.

The investigator must maintain an accurate record of the shipment and dispensing of study drug in a drug accountability ledger. Monitoring of drug accountability will be performed by the field monitor during site visits and at the completion of the trial. Patients will be asked to return all unused study drug and packaging at the end of the study or at the time of study drug discontinuation.

At the conclusion of the study, and as appropriate during the course of the study, the investigator will return all used and unused study drug, packaging, and a copy of the completed drug accountability ledger to the Novartis monitor or to the Novartis address provided in the investigator folder at each site.

6.5.4 Instructions for prescribing and taking the study drug

Investigator staff will instruct the patients to take the medication orally every morning either with or without food. The investigator should instruct the patient to take the study drug exactly as prescribed. The patient should be instructed to contact the investigator if he/she is unable for any reason to take the study drug as prescribed. Table 6-1 illustrates the instructions for study drug use.

The first dose of study medication will be taken on Baseline (Day 1) under the supervision of the study nurse. Dosing will be continued for 104 weeks until the Week 104 study visit. All study drug will be collected at the Week 104 visit and patients will **stop the study drug during the**

Post-treatment Phase of the study. Potential follow-up HBV treatment during this phase is based upon the discretion of the investigator and according to their clinical judgment and practice.

Compliance will be assessed by the investigator and/or study personnel at each visit using pill counts and information provided by the patient/caregiver. This information should be captured in the source document at each visit. Study personnel at clinical sites will be required to perform pill counts of study medication returned by patients at each clinic visit during the treatment period. Patients will be required to bring study medications with them for each study visit during the treatment period.

The investigator should promote compliance by instructing the patient to take the study drug exactly as prescribed and by stating that compliance is necessary for the patient’s safety and the validity of the study. The patient should be instructed to contact the investigator if he/she is unable for any reason to take the study drug as prescribed.

All dosages prescribed and dispensed to the patient and all dose changes during the study must be recorded on the Dosage Administration Record CRF (eCRF).

This document (090095af830c511d in docbase CREDI_EH) has been digitally signed with external signatures using Entrust PKI.
Signatures manifested as of 11/5/2011 6:28:07 AM, signing status at this time: Completed (1 of 1 signatures)
Approved for report publication by Poirier Kevin in East Hanover at Sat, 05 Nov 2011 02:19:57 AM EDT

6.5.5 Permitted study drug dose adjustments and interruptions

For patients who are unable to tolerate the tenofovir protocol-specified dosing scheme, the protocol allows for dose adjustments when renal insufficiency occurs transiently. Indicators of renal insufficiency include the following:

- 0.5 mg/dL increase in serum creatinine, confirmed on re-testing at the central study laboratory
- Estimated calculated creatinine clearance < 50 mL/minute, confirmed by two estimated calculations
- Laboratory serum phosphate, < 1.5 mg/dL, confirmed on re-testing at the central study laboratory

Transient renal insufficiency is defined by the presence of any of the above signs that show continued reduction in severity or disappear upon retesting. The tenofovir disoproxil fumarate (Viread®) package insert should guide dose adjustment for this treatment group. Patients who undergo dose adjustment should be monitored weekly for one month in order to document renal function. If creatinine clearance does not improve over one month, then the patient must be discontinued from study. If creatinine clearance is < 40 mL/min, at any time point in the study, then the patient must be discontinued from study.

These changes must be recorded on the Dosage Administration Record CRF (eCRF).

6.5.6 Rescue medication

No rescue antiviral therapy will be provided. In the event of premature patient withdrawal for insufficient therapeutic effect or viral breakthrough, or for ALT flare upon study completion at Week 104, the patient may be treated by the investigator according to local practice. The reason for such intervention will be clearly described in the eCRF (e.g., for insufficient therapeutic effect, safety). Patients who are discontinued or withdrawn from the study will have an Early Discontinuation Visit and then have a 30-day follow-up visit which will include all the assessments that are performed at the Week 104 visit.

6.5.7 Other concomitant treatment

Excluded concomitant treatments are as follows but are not limited to: dapsone, erythromycin, fluconazole, ketoconazole, rifampin, anti-tuberculosis regimens, others or nephrotoxic drugs (e.g. aminoglycosides, amphotericin B, foscarnet, vancomycin, cyclosporine, tacrolimus, or frequent NSAIDs or aspirin (administered daily for more than one week at a scheduled dose intended for anti-inflammatory therapy)). Please refer to Appendix 2 for further information on concomitant treatment.

The investigator should instruct the patient to notify the study site about any new medications he/she takes after the start of the study drug. All medications (other than study drug) and significant non-drug therapies (including physical therapy and blood transfusions) administered after the patient starts treatment with study drug must be listed on the Concomitant medications/Significant non-drug therapies section of the eCRF after start of study drug. Concomitant medications will be tabulated using World Health Organization (WHO) drug classifications. The number of patients using concomitant medications will be summarized by treatment group.

This document (090095af830c511d in docbase CREDI_EH) has been digitally signed with external signatures using Entrust PKI.
Signatures manifested as of 11/5/2011 6:28:07 AM, signing status at this time: Completed (1 of 1 signatures)
Approved for report publication by Poirier Kevin in East Hanover at Sat, 05 Nov 2011 02:19:57 AM EDT

6.5.8 Study drug discontinuation and premature patient withdrawal

Study drug discontinuation

Study treatment discontinuation is defined as discontinuation of telbivudine or tenofovir. Study drug must be discontinued for a given patient if the investigator determines that continuing it would result in a significant safety risk for that patient. The following circumstances **require** study drug discontinuation:

- Withdrawal of informed consent
- Patient enrolls in another clinical trial of an investigational agent while participating in this study
- Use of any of the prohibited concomitant medications indicated in Section 6.5.7 and Appendix 2
- Patient consistently fails to adhere to study drug regimen or protocol requirements leading to potential increased risk to the patient's safety
- Patient has clinical signs/symptoms of hepatic decompensation (see Table 6-2).
- Patient has cancer (other than basal cell or squamous cell carcinoma)
- Virologic breakthrough associated with evidence of disease progression (See Glossary of terms)
- Any of the following laboratory results:
  - Patient becomes co-infected with HCV, HDV, or HIV
  - Positive pregnancy test
  - Patient HBV DNA $\geq 3 \log_{10}$ copies/mL at Week 52
- Emergence of the following adverse events:
  - Patient has HCC
  - Nephrotoxicity demonstrated by an increase in serum creatinine: estimated calculated creatinine clearance by the Cockcroft-Gault method using ideal body weight below 40 mL/min. See Glossary of terms.
  - Severe ALT "flare" on treatment requiring hospitalization (See Glossary of terms)
  - Patients experiencing muscle pain on exam as indicated in Section 8.4

In addition to these requirements for study drug discontinuation, the investigator should discontinue study drug for a given patient if, on balance, he thinks that continuation would be detrimental to the patient's well-being.

Patients that discontinue study drug prior to Week 104 will have all the required assessments performed at Week 104 and the Visit 19 Treatment Study Phase Completion eCRF should be completed, giving the date and the primary reason for stopping the study drug. The patient will return for a 30-day follow-up visit that will include all the assessments that are performed at the Week 120 (A change in the completion guidelines for a select group of patients who would have their week 120 visit occur in the year 2012 is necessitated for the following reason: A critical assay that has been used throughout the entire study will no longer be available from the manufacturer. The current assay system will only remain valid until

This document (090095af830c511d in docbase CREDI_EH) has been digitally signed with external signatures using Entrust PKI.
Signatures manifested as of 11/5/2011 6:28:07 AM, signing status at this time: Completed (1 of 1 signatures)
Approved for report publication by Poirier Kevin in East Hanover at Sat, 05 Nov 2011 02:19:57 AM EDT

December 31, 2011. This unavailability of the HBV DNA assay will shorten the safety follow up period for patients whose final visit would occur in 2012. This change will reduce the follow up period for a select group of patients from 16 weeks to 12 weeks and will effect a maximum of 15 patients. The select group of patients correlates to those impacted by assay availability.) visit. Visits 20 through 22 will not be required.

Patients who discontinue study drug should NOT be considered withdrawn from the study. See Section 7 for the required assessments of these patients after study drug discontinuation.

Premature patient withdrawal

Patients may voluntarily withdraw from the study for any reason at any time. They may be considered withdrawn if they state an intention to withdraw, or fail to return for visits, or become lost to follow up for any other reason.

If premature withdrawal occurs for any reason, the investigator must determine the primary reason for a patient's premature withdrawal from the study and record this information with the date of study drug discontinuation on the Study Completion eCRF.

For patients who are lost to follow-up (i.e., those patients whose status is unclear because they fail to appear for study visits without stating an intention to withdraw), the investigator should show "due diligence" by documenting in the source documents steps taken to contact the patient, e.g., dates of telephone calls, registered letters, etc.

Patients who are prematurely withdrawn from the study will not be replaced.

Patients who prematurely withdrawal from study for any reason prior to week 104 will have all the required assessments performed at Week 104 and the Visit 19 Treatment Study Phase Completion eCRFs will be completed. The patient will return 30 days later for a follow-up visit that will include all the assessments that are performed at the Week 120 visit (A change in the completion guidelines for a select group of patients who would have their week 120 visit occur in the year 2012 is necessitated for the following reason: A critical assay that has been used throughout the entire study will no longer be available from the manufacturer. The current assay system will only remain valid until December 31, 2011. This unavailability of the HBV DNA assay will shorten the safety follow up period for patients whose final visit would occur in 2012. This change will reduce the follow up period for a select group of patients from 16 weeks to 12 weeks and will effect a maximum of 15 patients. The select group of patients correlates to those impacted by assay availability.). Visits 20 through 22 will not be required.

6.5.9 Nephrotoxicity

Chronic administration of TDF (300 mg once daily) may result in nephrotoxicity, especially in those patients with underlying chronic renal dysfunction and those taking concomitant nephrotoxic agents such as cyclosporine, tacrolimus, aminoglycosides, vancomycin and non-steroidal anti-inflammatory drugs. Overall, the risk of nephrotoxicity in patients with adequate renal function is low.

Changes in serum creatinine and serum phosphorus are the most specific markers for TDF-related nephrotoxicity. It is important to monitor renal function for all patients during treatment with TDF, particularly for those with pre-existing or other risks for renal

This document (090095af830c511d in docbase CREDI_EH) has been digitally signed with external signatures using Entrust PKI.
Signatures manifested as of 11/5/2011 6:28:07 AM, signing status at this time: Completed (1 of 1 signatures)
Approved for report publication by Poirier Kevin in East Hanover at Sat, 05 Nov 2011 02:19:57 AM EDT

impairment. In the event that a patient develops nephrotoxicity, treatment should be discontinued and appropriate intervention initiated according to local practice.

The pharmacokinetics of Viread®, and Sebivo® are altered in patients with renal impairment. It is important to monitor renal function for all patients during treatment with Viread®, and Sebivo®. For management of patients with renal insufficiency, please refer to the prescribing information for Viread® (Gilead Sciences, 2006) and Sebivo® (Idenix Pharmaceuticals, 2006). Patients that develop renal insufficiency while on study should be discontinued.

6.5.10 Signs of hepatic decompensation

Patients meeting post-baseline criteria for clinical or biochemical signs of hepatic decompensation must be withdrawn from the study (Refer to Table 6-2).

Table 6-2 Signs of hepatic decompensation

	Clinical and Biochemical Signs
Hepatic Decompensation	Clinically-evident ascites.
	Variceal or GI hemorrhage attributable to portal hypertension.
	Hepatic encephalopathy.
	Hepatorenal syndrome.
	Bacterial peritonitis and/or sepsis.
	Decrease in serum albumin to < 3.0 g/dL, confirmed on retesting at the central study laboratory, attributed to liver disease.
	Increase in serum bilirubin to ≥ 2 x ULN, confirmed on retesting at the central study laboratory, attributed to liver disease other than obstructive biliary tract disease.

Such findings should be documented on two study visits whenever possible, including off-schedule study visits if necessary.

Patients with hypophosphatemia with serum phosphate levels >1.5 mg/dL may remain on study. The low serum phosphate level must be confirmed on re-testing at the central laboratory within 3 working days if new finding since study entry. At the investigator's discretion, study medication may be held pending results. Phosphate supplementation with combination neutral phosphate solution, phosphate-rich food and fluids, or milk is strongly recommended.

6.5.11 Lack of efficacy at Week 24 or thereafter (persisting ALT elevation and evidence of insufficient viral response or Virologic Breakthrough)

After the first 24 weeks of study treatment, patients with persisting significant ALT elevation (e.g., ‡ 2 x ULN) and with serum HBV levels ≥ 3 $\log_{10}$ copies/mL can potentially represent: (a) a poor response to study treatment; or (b) virologic breakthrough with return of active necroinflammatory liver disease; or (c) noncompliance with medications, among other etiologies. These patients are not usually at risk for hepatic **decompensation** over short periods of time, but such patients should be discontinued from study if they meet either of the following two criteria outlined in Table 6-3.

Patients meeting either of the two criteria at Week 24 or thereafter, will be designated as treatment failures in the efficacy analyses and should be discontinued from the study. This

This document (090095af830c511d in docbase CREDI_EH) has been digitally signed with external signatures using Entrust PKI.
Signatures manifested as of 11/5/2011 6:28:07 AM, signing status at this time: Completed (1 of 1 signatures)
Approved for report publication by Poirier Kevin in East Hanover at Sat, 05 Nov 2011 02:19:57 AM EDT

will allow the election of other therapies for such patients, as deemed appropriate by the patient's clinicians.

Table 6-3 ALT elevation and insufficient viral response, or Virologic Breakthrough requiring withdrawal from study

Criterion	
Severe ALT elevation with HBV flare	At Week 24 or thereafter, ALT increases to 10 x ULN (and at least 2 x Baseline) on two or more visits over a period of at least 7 days AND serum HBV DNA is ≥ 3 log_{10} copies/mL OR the serum HBV DNA pattern meets the Virologic Breakthrough definition (described in Glossary of Terms)
Persisting moderate ALT elevation with HBV viremia	Over 16 weeks of study observation at any time at or after Week 24, ALT is persistently elevated to levels ≥ 2 x ULN (and ≥ Baseline ALT level) AND serum HBV DNA is ≥ 3 log_{10} copies/mL OR serum HBV DNA pattern meets the Virologic Breakthrough definition

Study drug discontinuation and withdrawal from the study for any reason is to be documented in the patient's source documents and on the Study Completion eCRF. Patients discontinuing from study for lack of efficacy are to be monitored clinically and via routine laboratory testing after discontinuation of treatment for 4 visits over 16 weeks utilizing the visit schedule for visits 20-23 , post treatment follow-up**.**

6.5.12 Emergency unblinding of treatment assignment

The study design will not have any treatment blinding, no emergency unblinding will be necessary.

6.5.13 Study completion and post-study treatment

Study completion is defined for an individual patient who meets the screen requirements, attends the Baseline Day 1 visit, completes the 104 week Treatment Phase of the study and 16 week Follow-up Phase of the study.

A change in the completion guidelines for a select group of patients who would have their week 120 visit occur in the year 2012 is necessitated for the following reason: A critical assay that has been used throughout the entire study will no longer be available from the manufacturer. The current assay system will only remain valid until December 31, 2011. This unavailability of the HBV DNA assay will shorten the safety follow up period for patients whose final visit would occur in 2012.

Study completion for those patients who are effected by this unavailability of the HBV DNA assay will be defined as those patients who have met the screening requirements, attended the Baseline Day 1 visit, completed the 104 week Treatment Phase of the study and the shortened 12 week Follow-up Phase of the study.

The study recruitment is completed when at least 100 patients have been assigned to the treatment group. Patients already in screening upon completion of enrollment should be allowed to enter the study if they meet all of the inclusion/exclusion criteria.

Patients who complete the treatment period enter the post-treatment follow-up phase. It is up to the investigator's discretion to decide on the requirement of additional HBV treatment

This document (090095af830c511d in docbase CREDI_EH) has been digitally signed with external signatures using Entrust PKI.
Signatures manifested as of 11/5/2011 6:28:07 AM, signing status at this time: Completed (1 of 1 signatures)
Approved for report publication by Poirier Kevin in East Hanover at Sat, 05 Nov 2011 02:19:57 AM EDT

based on local practice guidelines. The Sponsor will not provide drug therapy after the treatment period of 104 weeks. Any HBV treatment prescribed for the post-treatment follow-up phase must be recorded as concomitant medication.

The investigator also must provide follow-up medical care for all patients who are prematurely withdrawn from the study according to the local standard of care.

6.5.14 Early study termination

The study can be terminated at any time for any reason by Novartis Pharmaceuticals. Should this be necessary, the patient should be seen as soon as possible and treated as described in Section 7 for a prematurely withdrawn patient. The investigator may be informed of additional procedures to be followed in order to assure that adequate consideration is given to the protection of the patient's interests. The investigator will be responsible for informing IRBs and/or ECs of the early termination of the trial.

7 Visit schedule and assessments

Table 7-1 lists all the assessments from the Screening period through the Week 120 Post-treatment Follow-up period and indicates when each assessment is performed with an "X". Informed consent will be collected from all patients prior to entering into the Screening period. Consenting patients determined to be eligible during Screening will be assigned to the treatment arm prior to the first dose of study medication. Patients will return to the clinic according to the visit schedule outlined in Table 7-1 and the following assessments will be made:

- Medical history: To establish patient eligibility prior to treatment and to establish a baseline for safety reporting.
- Complete physical examination: To establish the overall health status of the patient at baseline, to supplement relevant medical history prior to treatment and to assess signs of AEs potentially unreported by the patient during treatment.
- Vital signs, body weight and height: To establish the overall health status of the patient at baseline, to supplement relevant medical history prior to treatment and to assess signs of AEs potentially unreported by the patient during treatment.
- HIV-1, HIV-2, HCV, and HDV DNA levels will be established for patient eligibility prior to treatment.
- If available, prior Liver Biopsy- pathology report of a biopsy performed within 24 months prior to Screening.
- AFP, amylase and lipase levels will be used to establish patient eligibility prior to treatment.
- HBeAg: To establish patient eligibility prior to treatment.
- HBeAb: To establish patient eligibility prior to treatment.
- HBsAg: To establish patient eligibility prior to treatment.
- HBsAb: To establish seroconversion during treatment.

This document (090095af830c511d in docbase CREDI_EH) has been digitally signed with external signatures using Entrust PKI.
Signatures manifested as of 11/5/2011 6:28:07 AM, signing status at this time: Completed (1 of 1 signatures)
Approved for report publication by Poirier Kevin in East Hanover at Sat, 05 Nov 2011 02:19:57 AM EDT

- HBV DNA level: To establish patient eligibility prior to treatment and to establish the impact of treatment.
- Pregnancy test: A serum pregnancy (β-HCG) test will be administered to women of childbearing potential during the Screening period to establish patient eligibility prior to treatment. Urine pregnancy tests will be administered at pre-assigned visits thereafter to ensure continued eligibility to remain on treatment.
- Biochemistry: Serum creatinine, serum phosphate, alanine aminotransferase (ALT), aspartate aminotransferase (AST), blood urea nitrogen (BUN), albumin (alb), total bilirubin (TB) and creatine kinease (CK): to establish patient eligibility and to assess safety.
- Prothrombin time (PT/INR) will be assessed to establish patient eligibility prior to treatment and to assess safety.
- Hematology: Hemoglobin (Hgb), hematocrit (Hct), platelets, white blood cell count (WBC), ANC will be performed to establish patient eligibility prior to treatment and to assess safety.
- Urinalysis (specific gravity, pH, protein, glucose and heme) will be performed to establish patient eligibility prior to treatment and to assess safety.
- ECGs (electrocardiogram): A twelve lead ECG will be collected at the baseline visit only for assessment of entry into the study. A record of the test and results should be stored in the source documents.

AEs will be recorded to monitor safety (see Section 7.5).

A serum sample will be collected at all study visits for storage. Samples may be used for repeat or supplemental laboratory analyses (e.g., for viral resistance mutation sequencing and repeat testing of specific analytes if the original sample is damaged or deemed unsuitable for analysis). The sample will be destroyed if no further testing is necessary.

Patients should be seen for all visits on the designated day or as close as possible to the scheduled visit day.

Patients who discontinue study drug before completing the study, and those who prematurely withdraw from the study for any reason, will have an Early Discontinuation Visit and then have a 30-day follow-up visit that will include all the assessments that are performed at the week 104 Treatment visit. If the patient refuses to return for these assessments or are unable to do so, every effort should be made to contact them or a knowledgeable informant by telephone to determine the reason for study discontinuation and the status of the patient.

At a minimum, patients discontinuing will be contacted for safety evaluations during the 30 days following the last dose of study drug, including final contact at the 30-day point. Documentation of attempts to contact the patient should be recorded in the patient record.

All data obtained from the assessments listed in Table 7-1 and described in detail in the subsections below must be supported in the patient's source documentation.

This document (090095af830c511d in docbase CREDI_EH) has been digitally signed with external signatures using Entrust PKI.
Signatures manifested as of 11/5/2011 6:28:07 AM, signing status at this time: Completed (1 of 1 signatures)
Approved for report publication by Poirier Kevin in East Hanover at Sat, 05 Nov 2011 02:19:57 AM EDT

Table 7-1 Assessment schedule

Study Phase:	Screen	Base line	Treatment (Week post-baseline)																Post-treatment Follow-up (Week post-baseline)			
Visit	1	2	3	4	5	6	7	8	9	10	11	12	13	14	15	16	17	18	19	20	21	22	23
Week		Day 1	2	4	8	12	16	24	26	30	40	48	52	60	68	76	86	96	104[c]	108	112	116	120[d]
Informed consent	X	X*																					
Inclusion/Exclusion	X	X*																					
Medical history	X																						
Complete physical	X												X						X				X
Vital signs	X	X	X	X	X	X	X	X	X	X	X	X	X	X	X	X	X	X	X	X	X	X	X
Body wt. & height[a]	X												X						X				X
HBV Genotype	~~X~~	**X**																					
HBV Sequencing												X							X				
HIV-1, HIV-2, HCV, HDV	X																						
AFP (screen only), amylase, lipase	X	X						X					X						X				
HBeAg/HBeAb	X	X											X						X				X
HBsAg/HBsAb	X	X											X						X				X
HBV DNA	X	X	X	X	X	X	X	X		X	X	X	X	X	X	X	X	X	X	X	X	X	X
Pregnancy test[b]	X	X	X	X	X	X	X	X		X	X	X	X	X	X	X	X	X	X				
Biochemistry	X	X	X	X	X	X	X	X		X	X	X	X	X	X	X	X	X	X	X	X	X	X
Hematology	X					X		X			X		X		X		X		X		X		X
Prothrombin time	X																						
Serum for storage	X	X	X	X	X	X	X	X		X	X	X	X	X	X	X	X	X	X	X	X	X	X
Urinalysis	X	X	X	X	X	X		X					X						X				

This document (090095af830c511d in docbase CREDI_EH) has been digitally signed with external signatures using Entrust PKI.
Signatures manifested as of 11/5/2011 6:28:07 AM, signing status at this time: Completed (1 of 1 signatures)
Approved for report publication by Poirier Kevin in East Hanover at Sat, 05 Nov 2011 02:19:57 AM EDT

Study Phase:	Screen	Base line	Treatment (Week post-baseline)																	Post-treatment Follow-up (Week post-baseline)			
Visit	1	2	3	4	5	6	7	8	9	10	11	12	13	14	15	16	17	18	19	20	21	22	23
Week		Day 1	2	4	8	12	16	24	26	30	40	48	52	60	68	76	86	96	104[c]	108	112	116	120[d]
ECG	X																						
AEs		X	X	X	X	X	X	X	X	X	X	X	X	X	X	X	X	X	X	X	X	X	X
Dispense telbivudine study medication		X		X	X	X	X			X	X	X	X	X	X	X	X	X					
Dispense tenofovir study medication (IF HBV DNA ≥ 300 copies/ml at Wk 24)									X	X	X	X	X	X	X	X	X	X					

* Confirm at Baseline Visit; [a] Screening only; [b]Screening serum pregnancy test results will be recorded in the database, all urine pregnancy test results will be recorded in the patient source documents. [c]Week 104 or early Treatment Phase Discontinuation visit; [d]Week 120 or early Post-Treatment Follow-up Phase Discontinuation visit ((A change in the completion guidelines for a select group of patients who would have their week 120 visit occur in the year 2012 is necessitated for the following reason: A critical assay that has been used throughout the entire study will no longer be available from the manufacturer. The current assay system will only remain valid until December 31, 2011. This unavailability of the HBV DNA assay will shorten the safety follow up period for patients whose final visit would occur in 2012. This change will reduce the follow up period for a select group of patients from 16 weeks to 12 weeks and will effect a maximum of 15 patients. The select group of patients correlates to those impacted by assay availability.)

This document (090095af830c511d in docbase CREDI_EH) has been digitally signed with external signatures using Entrust PKI.
Signatures manifested as of 11/5/2011 6:28:07 AM, signing status at this time: Completed (1 of 1 signatures)
Approved for report publication by Poirier Kevin in East Hanover at Sat, 05 Nov 2011 02:19:57 AM EDT

7.1 Information to be collected on screening failures

Patient demographics will be collected for screen failures. This will be recorded for all screen-failure patients along with the reason for screen failure. All other information will be retained with the patient's study source documentation.

7.2 Patient demographics/other baseline characteristics

Patient race will be collected as one of the following:

- Caucasian
- Black
- Asian
- Native American
- Pacific Islander
- Other (specify)

Patient baseline characteristics that will determine their eligibility for participation in the study will be based on the following laboratory assessments (see inclusion criteria):

- Medical history
- Complete physical examination
- Vital signs and body weight and height
- HIV-1, HIV-2, HCV, and HDV DNA levels
- AFP, amylase and lipase levels
- HBeAg/ HBeAb
- HBsAg/HBsAb
- HBV DNA level
- Pregnancy test
- Biochemistry
- Hematology
- Urinalysis
- ECG

7.3 Treatment exposure and compliance

Compliance will be assessed by the investigator and/or study personnel at each visit using pill counts and information provided by the patient. This information should be captured in the source document at each visit. Study personnel at clinical sites will be required to perform pill counts of study medication returned by patients at each clinic visit during the treatment period. Patients will be required to bring study medications with them for each study visit during the treatment period.

The investigator should promote compliance by instructing the patient to take the study drug exactly as prescribed and by stating that compliance is necessary for the patient's safety and

This document (090095af830c511d in docbase CREDI_EH) has been digitally signed with external signatures using Entrust PKI.
Signatures manifested as of 11/5/2011 6:28:07 AM, signing status at this time: Completed (1 of 1 signatures)
Approved for report publication by Poirier Kevin in East Hanover at Sat, 05 Nov 2011 02:19:57 AM EDT

the validity of the study. The patient should be instructed to contact the investigator if he/she is unable for any reason to take the study drug as prescribed.

7.4 Efficacy

7.4.1 Serum HBV DNA Assessments

Serum HBV DNA determinations will be performed at a central reference laboratory through use of the COBAS Amplicor HBV Monitor assay (Roche Molecular Systems, Branchburg, NJ, USA), which utilizes polymerase chain reaction (PCR) methods and semi-automated sample readout technologies (threshold for detection 300 copies/mL). Pre-dilution of serum samples will be utilized at the central laboratory, to accommodate the range of HBV viremia commonly found in patients with CHB and active virus replication (e.g., 6-9 log_{10} copies/mL). Diluted samples found to be negative for HBV DNA will be retested undiluted. The COBAS Amplicor HBV Monitor assay has been used in the previously conducted Phase II and Phase III telbivudine studies, and thus the results from this study will be able to be linked to existing data on telbivudine.

Serum samples for HBV DNA will be obtained during Screening to determine eligibility for the study. The Screening serum HBV DNA values must be ‡ 5 log_{10} copies/mL by the COBAS Amplicor HBV Monitor assay.

Serum samples for HBV DNA will be obtained during Screening and the Baseline Visit (Day 1), and at every protocol-stipulated Treatment Phase study visit (See Table 7-1), and at the Early Treatment Discontinuation visit if applicable.

7.4.2 Serologic Markers

Other HBV serologic markers (HBsAg/Ab, HBeAg/Ab) will be assessed at the central laboratory using standard commercially-available enzyme immunoassays. Serum for HBsAg/Ab and HBeAg/Ab will be obtained according to the schedule in Table 7-1 or at the Early Treatment Discontinuation Visit.

7.4.3 ALT

Serum ALT normalization is an important clinically-relevant efficacy endpoint. Elevated serum ALT levels are thought to reflect underlying hepatitis disease activity (i.e., active liver inflammation). Correspondingly, ALT normalization is an accepted therapeutic goal in hepatitis studies as it is thought to reflect a substantial reduction in hepatic disease activity. ALT levels will be determined from serum samples obtained at all study visits, via standard central laboratory testing.

7.5 Safety

7.5.1 Physical examination

Investigator will perform a complete physical examination during the visits indicated on Table 7-1, at the Early Treatment Discontinuation visit, as necessary for AEs or patient requests. A complete physical may include, but is not limited to the following: the examination of general appearance, skin, neck (including thyroid), eyes, ears, nose, throat, lungs, heart, abdomen,

This document (090095af830c511d in docbase CREDI_EH) has been digitally signed with external signatures using Entrust PKI.
Signatures manifested as of 11/5/2011 6:28:07 AM, signing status at this time: Completed (1 of 1 signatures)
Approved for report publication by Poirier Kevin in East Hanover at Sat, 05 Nov 2011 02:19:57 AM EDT

back, rectal (optional), external genitalia (optional), lymph nodes, extremities, vascular and neurological system.

Information for all physical examinations must be included in the source documentation at the study site. Significant findings that are present prior to the start of study drug must be included in the Relevant Medical History/Current Medical Conditions screen on the patient's eCRF. Significant findings made after the start of study drug which meet the definition of an Adverse Event must be recorded on the Adverse Event screen of the patient's eCRF.

7.5.2 Vital signs

The investigator will obtain vital signs (heart rate, blood pressure, temperature and respirations), body weight and height during the visits indicated on Table 7-1 and at the Early Treatment Discontinuation visit, as necessary for AEs or patient request.

7.5.3 Height and weight

Height in centimeters (cm) will be measured during the visits indicated on Table 7-1 and at the Early Treatment Discontinuation visit, as necessary for AEs or patient request.

Body weight (to the nearest 0.1 kilogram [kg] in indoor clothing, but without shoes) will be measured during the visits indicated on Table 7-1 and at the Early Treatment Discontinuation visit, as necessary for AEs or patient request.

7.5.4 Laboratory evaluations

The investigator will collect blood serum for the following tests according to the visit schedule on Table 7-1 and at the Early Treatment Discontinuation visit if applicable: HIV-1, HIV-2, HCV and HDV DNA levels, AFP, amylase, lipase, HBeAg/Ab, HBsAg/Ab, biochemistry including CK, hematology panels and prothrombin time (PT/INR). A urinalysis, including heme with reflex myoglobin testing as needed will be collected at visits indicated on Table 7-1 to measure specific gravity, pH, protein and glucose.

7.5.5 Electrocardiogram (ECG)

A standard 12 lead ECG will be performed locally for all patients during the Screening visit. Interpretation of the tracing must be made by a qualified physician and documented on the ECG section of the eCRF. Each ECG tracing should be labeled with the study number, patient initials, patient number, date, and kept in the source documents at the study site. Clinically significant abnormalities should be recorded on the relevant Medical history/Current medical conditions eCRF page. Clinically significant findings must be discussed with the Novartis Medical Monitor prior to enrolling the patient in the study.

7.5.6 Pregnancy and assessments of fertility

β-HCG testing for pregnancy for all women of child-bearing potential will be collected at the Screening visit. Thereafter, at each schedule visit on Table 7-1, a urine pregnancy test will be completed and at the Early Treatment Discontinuation visit if applicable. If positive test results are obtained, the patient must be discontinued from the trial.

This document (090095af830c511d in docbase CREDI_EH) has been digitally signed with external signatures using Entrust PKI.
Signatures manifested as of 11/5/2011 6:28:07 AM, signing status at this time: Completed (1 of 1 signatures)
Approved for report publication by Poirier Kevin in East Hanover at Sat, 05 Nov 2011 02:19:57 AM EDT

7.5.7 Unscheduled safety visits

Unscheduled safety visits will be permitted at any time if deemed clinically necessary by the investigator. Should the patient require an unscheduled safety visit, the study staff will measure vital signs, record adverse events, concomitant medications if applicable and perform those evaluations deemed clinically necessary for the visit.

7.6 Other assessments

No additional tests will be performed on patients entered into this study.

8 Safety monitoring

8.1 Adverse events

An adverse event is the appearance or worsening of any undesirable sign, symptom, or medical condition occurring after starting the study drug even if the event is not considered to be related to study drug. Study drug includes the investigational drug under evaluation and the comparator drug or placebo that is given during any phase of the study. Medical conditions/diseases present before starting study drug are only considered adverse events if they worsen after starting study drug. Abnormal laboratory values or test results constitute adverse events only if they induce clinical signs or symptoms, are considered clinically significant, or require therapy.

The occurrence of adverse events should be sought by non-directive questioning of the patient at each visit during the study. Adverse events also may be detected when they are volunteered by the patient during or between visits or through physical examination, laboratory test, or other assessments. All adverse events must be recorded on the Adverse Events CRF with the following information:

1. the severity grade (mild, moderate, severe)
2. its relationship to the study drug(s) (suspected/not suspected)
3. its duration (start and end dates or if continuing at final exam)
4. whether it constitutes a serious adverse event (SAE)

An SAE is defined as an event which:

- is fatal or life-threatening
- results in persistent or significant disability/incapacity
- constitutes a congenital anomaly/birth defect
- requires inpatient hospitalization or prolongation of existing hospitalization, unless hospitalization is for:
  - routine treatment or monitoring of the studied indication, not associated with any deterioration in condition
  - elective or pre-planned treatment for a pre-existing condition that is unrelated to the indication under study and has not worsened since the start of study drug

This document (090095af830c511d in docbase CREDI_EH) has been digitally signed with external signatures using Entrust PKI.
Signatures manifested as of 11/5/2011 6:28:07 AM, signing status at this time: Completed (1 of 1 signatures)
Approved for report publication by Poirier Kevin in East Hanover at Sat, 05 Nov 2011 02:19:57 AM EDT

- treatment on an emergency outpatient basis for an event not fulfilling any of the definitions of a SAE given above and not resulting in hospital admission
- social reasons and respite care in the absence of any deterioration in the patient's general condition
- is medically significant, i.e. defined as an event that jeopardizes the patient or may require medical or surgical intervention to prevent one of the outcomes listed above

Unlike routine safety assessments, SAEs are monitored continuously and have special reporting requirements; see Section 8-2.

All adverse events should be treated appropriately. Treatment may include one or more of the following: no action taken (i.e. further observation only); study drug dosage adjusted/temporarily interrupted; study drug permanently discontinued due to this adverse event; concomitant medication given; non-drug therapy given; patient hospitalized/patient's hospitalization prolonged. The action taken to treat the adverse event should be recorded on the Adverse Event CRF.

Once an adverse event is detected, it should be followed until its resolution or until it is judged to be permanent, and assessment should be made at each visit (or more frequently, if necessary) of any changes in severity, the suspected relationship to the study drug, the interventions required to treat it, and the outcome.

Information about common side effects already known about the investigational drug can be found in the Investigator Brochure (IB) or will be communicated between IB updates in the form of Investigator Notifications. This information will be included in the patient informed consent and should be discussed with the patient during the study as needed.

8.2 Serious adverse event reporting

To ensure patient safety, every SAE, regardless of suspected causality, occurring after the patient has begun treatment and until 4 weeks after the patient has stopped study participation must be reported to Novartis within 24 hours of learning of its occurrence.

Any SAEs experienced after this 4-week period should only be reported to Novartis if the investigator suspects a causal relationship to the study drug.

Recurrent episodes, complications, or progression of the initial SAE must be reported as follow-up to the original episode, regardless of when the event occurs. This report must be submitted within 24 hours of the investigator receiving the follow-up information. An SAE that is considered completely unrelated to a previously reported one should be reported separately as a new event.

Information about all SAEs is collected and recorded on the Serious Adverse Event Report Form. The investigator must assess the relationship to study drug, complete the SAE Report Form in English, and send the completed, signed form by fax within 24 hours to the local Novartis Integrated Medical Safety Department. The telephone and telefax number of the contact persons in the local department of Integrated Medical Safety, specific to the site, are listed in the investigator folder provided to each site. The original copy of the SAE Report Form and the fax confirmation sheet must be kept with the case report form documentation at the study site.

This document (090095af830c511d in docbase CREDI_EH) has been digitally signed with external signatures using Entrust PKI.
Signatures manifested as of 11/5/2011 6:28:07 AM, signing status at this time: Completed (1 of 1 signatures)
Approved for report publication by Poirier Kevin in East Hanover at Sat, 05 Nov 2011 02:19:57 AM EDT

Follow-up information is sent to the same person to whom the original SAE Report Form was sent, using a new SAE Report Form stating that this is a follow-up to the previously reported SAE and giving the date of the original report. The follow-up information should describe whether the event has resolved or continues, if and how it was treated, whether the blind was broken or not, and whether the patient continued or withdrew from study participation.

If the SAE is not previously documented in the Investigator's Brochure or Package Insert (new occurrence) and is thought to be related to the Novartis study drug, an Integrated Medical Safety Department associate may urgently require further information from the investigator for Health Authority reporting. Novartis may need to issue an Investigator Notification (IN) to inform all investigators involved in any study with the same drug that this SAE has been reported. Suspected Unexpected Serious Adverse Reactions (SUSARs) will be collected and reported to the competent authorities and relevant ethics committees in accordance with Directive 2001/20/EC or as per national regulatory requirements in participating countries.

8.3 Pregnancies

To ensure patient safety, each pregnancy in a patient on study drug must be reported to Novartis within 24 hours of learning of its occurrence. The pregnancy should be followed up to determine outcome, including spontaneous or voluntary termination, details of the birth, and the presence or absence of any birth defects, congenital abnormalities, or maternal and/or newborn complications.

Pregnancy should be recorded on a Clinical Trial Pregnancy Form and reported by the investigator to the local Novartis Clinical Safety & Epidemiology Department. Pregnancy follow-up should be recorded on the same form and should include an assessment of the possible relationship to the Novartis study drug of any pregnancy outcome. Any SAE experienced during pregnancy must be reported on the SAE Report Form.

Pregnancy outcomes must be collected for the female partners of any males who took study drug in this study. Consent to report information regarding these pregnancy outcomes should be obtained from the mother.

8.4 Adverse events of special interest (AESIs) reporting

For the purpose of this study, adverse events of special interest (AESI) include muscle weakness, myopathy or myositis.

All AESI's should be recorded on the AESI Report Form and must be reported via FAX to the Novartis Global Clinical Trial Leader. The telephone and telefax number of the contact persons will be listed on the AESI document. The original copy of the AESI Report Form and the fax confirmation sheet must be kept with the source documentation at the study site.

Patients reporting such events should be managed according to the Muscle Symptom Algorithm provided in this protocol Section 8.4.1.

Follow-up information should be sent to the same person to whom the original AESI Report Form was sent, using a new AESI Report form stating that it is a follow-up to the previously reported AESI and giving the date of the original report. The follow-up information should

This document (090095af830c511d in docbase CREDI_EH) has been digitally signed with external signatures using Entrust PKI.
Signatures manifested as of 11/5/2011 6:28:07 AM, signing status at this time: Completed (1 of 1 signatures)
Approved for report publication by Poirier Kevin in East Hanover at Sat, 05 Nov 2011 02:19:57 AM EDT

describe whether the event has resolved or continues, if and how it was treated, and whether the patient continued or withdrew from study participation.

8.4.1 Clinical trial subjects with musculoskeletal complaints in Clinical Trial Subjects with Musculoskeletal Complaints

Subjects participating in this telbivudine clinical trial with complaints of muscle weakness, muscle pain or achiness, or effort-related fatigue should be managed according to the following algorithm. All subjects being followed with this algorithm should have a physical exam with muscle strength testing and Muscle Symptom Questionnaire administered at each visit.

MUSCLE SYMPTOM ALGORITHM

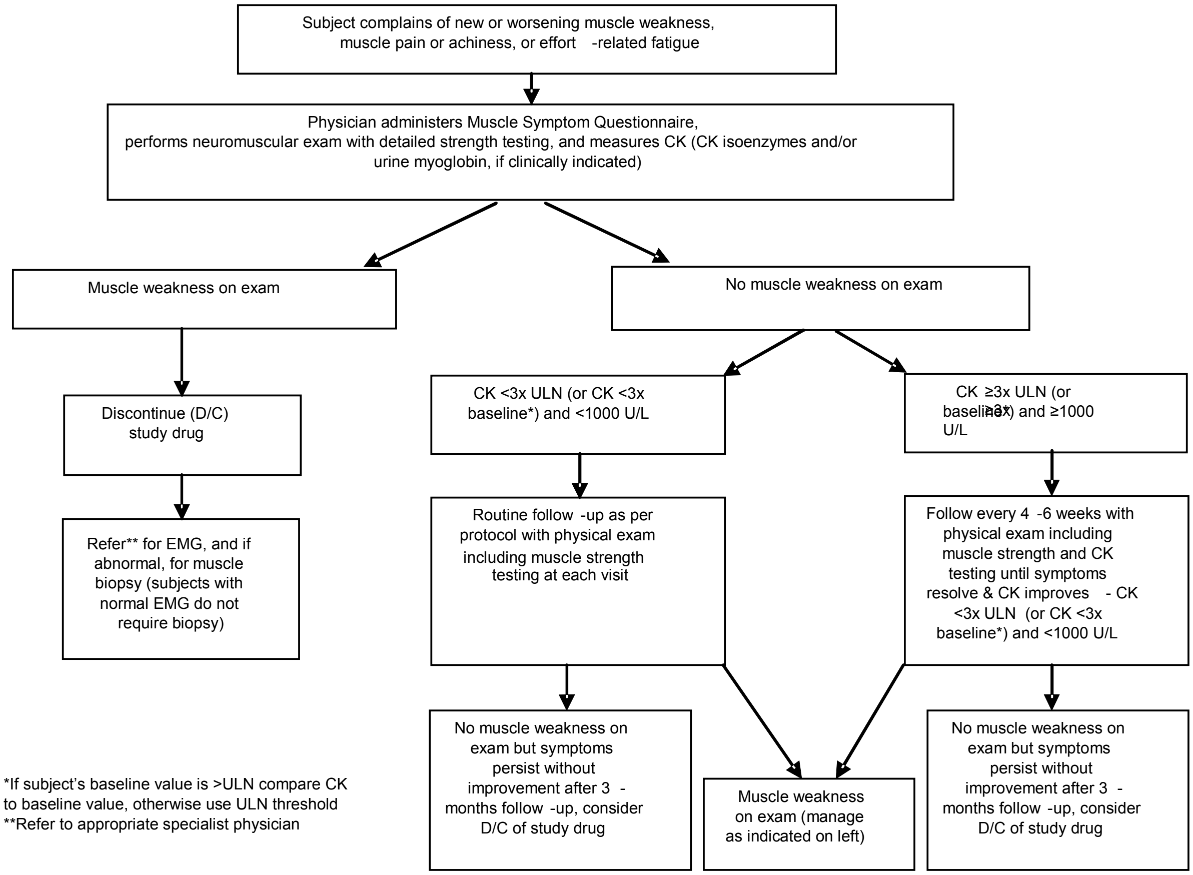

Subjects with Elevated CK

As stated above, subjects with muscle symptoms and elevated CK in the absence of muscle weakness should be followed every 4-6 weeks with a physical exam (including muscle strength testing) and CK assessment until CK level improves or normalizes or symptoms improve or resolve. Clinical trial subjects with muscle symptoms and lower level CK elevations will have routine follow-up per study protocol with the addition of a physical exam (with muscle strength testing). Subjects with evidence of muscle weakness on exam should be managed according to the algorithm on the left side of the diagram.

This document (090095af830c511d in docbase CREDI_EH) has been digitally signed with external signatures using Entrust PKI.
Signatures manifested as of 11/5/2011 6:28:07 AM, signing status at this time: Completed (1 of 1 signatures)
Approved for report publication by Poirier Kevin in East Hanover at Sat, 05 Nov 2011 02:19:57 AM EDT

Muscle Biopsy Protocol

Proper performing and processing of the muscle biopsies require open biopsies snap-frozen in isopentane, cooled in liquid nitrogen. Frozen sections are then processed for muscle enzyme histochemistry and immunocytochemistry (if needed). This is the only way to exclude biochemical or enzymatic defects and, in cases of polymyositis, to exclude Inclusion Body Myositis.

Confirmation of polymyositis in non-typical cases through the identification of CD8-positive cells and MHC-class I expression, hallmarks of polymyositis, can only be performed in frozen sections. All biopsies will be performed locally.

Definition of Myopathy

Myopathy: objective evidence of muscle weakness (proximal or distal) with or without pain that persists for greater than 4 weeks AND elevated CK ≥3x ULN.

Muscle Symptom Questionnaire

The Muscle Symptom Questionnaire (provided by Novartis) must be administered to all subjects who complain of new or worsening muscle weakness, muscle pain or achiness, or effort-related fatigue. This questionnaire will be provided to all study sites as appendix 3 and appendix 4 to this protocol and will be recorded in the study eCRFs.

8.5 Data Monitoring Committee

No data monitoring board is planned for this study.

9 Data review and database management

9.1 Site monitoring

Each investigator must submit an informed consent form to the Institutional Review Board (IRB)/Independent Ethics Committee (IEC) for their review and approval before enrolling patients. A copy of the EC approval of the informed consent form must be forwarded to Novartis or its designee, before initiation of the study. Novartis will maintain a copy of the approved informed consent form. Informed consent must be obtained from each patient or their legal guardian before initiation of protocol-specified study procedures and enrollment in the study. Each investigator must retain the original signed informed consent form. A copy of the signed informed consent form will be given to the patient. The investigator will not undertake any investigation specifically required by the protocol until valid informed consent has been obtained. The date obtained must be documented in the eCRF as well as the source document. If a protocol amendment is made, then the informed consent form must be revised to reflect the changes made to the protocol. After receiving EC approval, the revised informed consent form must be signed by patients currently participating in the study and also by potential patients before initiation of study procedures

Before study initiation, at a site initiation visit or at an investigator's meeting, a Novartis representative will review the protocol and (e)CRFs with the investigators and their staff.

This document (090095af830c511d in docbase CREDI_EH) has been digitally signed with external signatures using Entrust PKI.
Signatures manifested as of 11/5/2011 6:28:07 AM, signing status at this time: Completed (1 of 1 signatures)
Approved for report publication by Poirier Kevin in East Hanover at Sat, 05 Nov 2011 02:19:57 AM EDT

During the study, the field monitor will visit the site regularly to check the completeness of patient records, the accuracy of entries on the (e)CRFs, the adherence to the protocol and to Good Clinical Practice, the progress of enrollment, and to ensure that study drug is being stored, dispensed, and accounted for according to specifications. Key study personnel must be available to assist the field monitor during these visits. Before study initiation, at a site initiation visit or at an investigator's meeting, a Novartis representative will review the protocol and (e)CRFs with the investigators and their staff. During the study, the field monitor will visit the site regularly to check the completeness of patient records, the accuracy of entries on the (e)CRFs, the adherence to the protocol and to Good Clinical Practice, the progress of enrollment, and to ensure that study drug is being stored, dispensed, and accounted for according to specifications. Key study personnel must be available to assist the field monitor during these visits.

The investigator must maintain source documents for each patient in the study, consisting of case and visit notes (hospital or clinic medical records) containing demographic and medical information, laboratory data, electrocardiograms, and the results of any other tests or assessments. All information on (e)CRFs must be traceable to these source documents in the patient's file. Data not requiring a separate written record will be defined before study start and will be recorded directly on the CRFs. The investigator must also keep the original informed consent form signed by the patient (a signed copy is given to the patient).

The investigator must give the monitor access to all relevant source documents to confirm their consistency with the (e)CRF entries. Novartis monitoring standards require full verification for the presence of informed consent, adherence to the inclusion/exclusion criteria, documentation of SAEs, and the recording of data that will be used for all primary and safety variables. Additional checks of the consistency of the source data with the (e)CRFs are performed according to the study-specific monitoring plan. No information in source documents about the identity of the patients will be disclosed.

9.2 Data collection

Novartis will supply the investigator site with a computer loaded with Electronic Data Capture (EDC) software that has been fully validated and conforms to 21 CFR Part 11 requirements. Novartis personnel will train designated investigator site staff on the EDC system. Investigator site staff will not be given access to the EDC system until they have been trained. Designated investigator staff will enter the data required by the protocol into the Novartis eCRFs using the Novartis-supplied computer. Automatic validation programs check for data discrepancies in the eCRFs and, by generating appropriate error messages, allow the data to be confirmed or corrected by the investigator staff before transfer of the data to Novartis via a secure Virtual Private Network. The investigator must certify that the data entered are complete and accurate by signing a memo generated at the end of the trial that will be sent to him by Novartis personnel. After database lock, the investigator will receive a CD-ROM or paper copies of the patient data for archiving at the investigational site.

9.3 Database management and quality control

Novartis staff review the data entered into the eCRFs by investigational staff for completeness and accuracy and instruct the site personnel to make any required corrections or additions.

This document (090095af830c511d in docbase CREDI_EH) has been digitally signed with external signatures using Entrust PKI.
Signatures manifested as of 11/5/2011 6:28:07 AM, signing status at this time: Completed (1 of 1 signatures)
Approved for report publication by Poirier Kevin in East Hanover at Sat, 05 Nov 2011 02:19:57 AM EDT

Obvious errors are corrected by Novartis personnel. Queries are sent to the investigational site using an electronic data query. Designated investigator site staff is required to respond to the query and confirm or correct the data. If the electronic query system is not used, a paper Data Query Form will be faxed to the site. Site personnel will complete and sign the faxed copy and fax it back to Novartis staff who will make the correction to the database. The signed copy of the Data Query Form is kept at the investigator site.

Concomitant medications entered into the database will be coded using the WHO Drug Reference List, which employs the Anatomical Therapeutic Chemical classification system. Medical history/current medical conditions and adverse events will be coded using the Medical dictionary for regulatory activities (MedDRA) terminology.

Laboratory samples will be processed centrally and the results will be sent electronically to Novartis (or a designated CRO).

10 Data analysis

Data will be analyzed when all patients complete 52 weeks of study (primary analyses), and complete Post-treatment Follow-up period (120 weeks, final analysis). It is planned that data from all centers that participate in this protocol will be pooled, so that an adequate number of patients will be available for analysis.

When appropriate, separate summaries will be generated for patients with add on tenofovir (Week 24 $\geq$ 300 copies/ml: LDT+TDF) and patients continuing on telbivudine (Week 24 $<$ 300 copies/ml: LDT). There is no intention to compare the two subgroups by means of statistical hypothesis testing. However, statistical tests to assess within-group changes from baseline will be performed. In addition to summaries by these two groups, summary statistics for the two groups combined will also be presented.

10.1 Populations for analysis

The **ITT population** will consist of all patients who received at least one dose of study drug and have at least one post-baseline assessment of serum HBV DNA. Efficacy analyses will be conducted over the ITT population and following the intent-to-treatment principle.

The **Per-protocol population** will be defined as a subset of ITT population who do not have major protocol deviations, such as patient has poor compliance or patient takes prohibited medications during treatment period. Other criteria for major protocol deviations will be established and documented based upon the review of data before clinical database lock for the week 52 primary data analyses.

The **Safety Population** will consist of all patients who received at least one dose of study drug and had at least one post-baseline safety assessment. Patients will be analyzed according to treatment received and overall. Safety analysis will be performed on the safety population.

10.2 Patient demographics/other baseline characteristics

Demographic and baseline characteristics will be summarized for the ITT population by treatment groups and overall. Continuous variables will be presented with mean, median, standard deviation, minimum and maximum, and the number of non-missing observations.

This document (090095af830c511d in docbase CREDI_EH) has been digitally signed with external signatures using Entrust PKI.
Signatures manifested as of 11/5/2011 6:28:07 AM, signing status at this time: Completed (1 of 1 signatures)
Approved for report publication by Poirier Kevin in East Hanover at Sat, 05 Nov 2011 02:19:57 AM EDT

Categorical data will be displayed via absolute and relative frequencies for each category (including a category labeled as 'missing' when appropriate).

10.3 Treatments (study drug, rescue medication, other concomitant therapies, compliance)

The numbers of patients who receive each treatment and the duration of exposure will be summarized for the ITT population. In calculation of duration, periods of temporary interruption of study medication for safety reasons will be included. Further, frequency of dose change (including temporary dose interruption) by reasons will be presented.

Prior and concomitant therapies will be listed. The frequency and percentage of patients who used prior or concomitant medication will be summarized by preferred term (WHO Drug) and treatment group. Concomitant medications will also be checked for protocol deviations. Patients who took prohibited concomitant medications will be noted in the summary of protocol deviations.

The Investigator will assess the patient's compliance for each visit during treatment. Patients' compliance will be summarized by visit.

10.4 Analysis of the primary objective(s)

The primary objective of the study is to demonstrate the antiviral efficacy of telbivudine lead-in therapy for 24 weeks with or without treatment intensification with tenofovir disoproxil fumarate in adult patients with HBeAg-positive chronic hepatitis B (CHB) as demonstrated by HBV DNA non-detectability at week 52.

10.4.1 Variable

Primary efficacy endpoint is HBV DNA non-detectability at week 52.

10.4.2 Statistical method of analysis

To evaluate the primary objective, the number and proportion of patients (response rate) who achieve HBV DNA non-detectability will be estimated along with a 95% confidence interval at week 52. The 95% confidence interval is calculated as follows:

(1) $$\hat{P} - Z_a * \sqrt{\hat{P}(1 - \hat{P})/n}$$

where $\hat{P}$ is the estimated sample response rate of HBV DNA non-detectability;

$Z_a = F^{-1}(1 - a)$ with F the standard normal distribution; and

n is the sample size.

10.4.3 Handling of missing values/censoring/discontinuations

HBV DNA assay results below the lower limit of detection (LLD) of the assay of laboratory performing the test will be assigned a value equal to one half of the LLD for the statistical analyses and tabulations. For patients with missing baseline laboratory results, the baseline value will be imputed from the Screening value. All baseline change calculations are based on

This document (090095af830c511d in docbase CREDI_EH) has been digitally signed with external signatures using Entrust PKI.
Signatures manifested as of 11/5/2011 6:28:07 AM, signing status at this time: Completed (1 of 1 signatures)
Approved for report publication by Poirier Kevin in East Hanover at Sat, 05 Nov 2011 02:19:57 AM EDT

the difference from baseline. The last on-treatment observation carried forward method (LOCF) will be applied for on-treatment missing values.

When patients meet the protocol defined criteria for treatment discontinuation for efficacy and subsequently discontinue treatment, their post-treatment efficacy endpoint values will not be used for any on-treatment analysis (analysis done on scheduled treatment visit: week 1 to week 104). For these on-treatment analyses, the last on-treatment assessment will be carried forward for any efficacy observation occurring after the patient discontinues treatment due to efficacy.

For the analysis of the primary efficacy endpoint, all patients in the ITT population will be included.

10.4.4 Supportive analyses

Per-protocol analysis

As a supportive analysis and robustness check of primary analysis, the analysis outlined in Section 10.4.2 will be repeated for Per-protocol population, when >5% of patients have major protocol deviations (pre-defined before week 52 database lock for the primary analyses).

Rates and Confidence Intervals by visit

For primary efficacy endpoint, the response rates and 95% confidence intervals are calculated for each visit and displayed by both table and graph. The analyses are based on ITT population and Per-protocol population respectively.

In addition, a subgroup analysis for patients with baseline ALT $\geq 2 \times$ ULN and patients with baseline $5 \leq$ HBV DNA $< 9 \log_{10}$ copies per ml will also be performed.

10.5 Analysis of secondary objectives

10.5.1 Efficacy (secondary)

All secondary efficacy analyses will be performed on the ITT population. The key secondary objectives of the study are:

- To estimate the rate of virologic breakthrough up to week 48 and week 104
- To assess the rate of treatment-emergent genotypically confirmed HBV resistance associated with viral breakthrough up to weeks 48 and 104

Other secondary objectives of the study include:

- Assessment of HBV DNA non-detectability, reduction in HBV DNA from baseline and sustained reduction in HBV DNA over the course of the study
- Assessment of HBeAg loss and HBeAg seroconversion (defined as loss of HBeAg and development of HBeAb) over course of study
- To describe the ALT normalization rate at weeks 52 and 104

The secondary endpoints can be grouped into three categories and all endpoints belong to a same category will be analyzed using the same approach.

This document (090095af830c511d in docbase CREDI_EH) has been digitally signed with external signatures using Entrust PKI.
Signatures manifested as of 11/5/2011 6:28:07 AM, signing status at this time: Completed (1 of 1 signatures)
Approved for report publication by Poirier Kevin in East Hanover at Sat, 05 Nov 2011 02:19:57 AM EDT

In addition, subgroup analyses will be performed for all secondary efficacy endpoints for patients with baseline ALT ≥ 2 × ULN and those with baseline 5 ≤ HBV DNA < 9 $\log_{10}$ copies per ml.

Continuous endpoints

Continuous endpoints include serum HBV DNA in log copies/mL and serum ALT. Summary statistics and summary statistics of change from baseline, including means, standard deviations, medians, 25% percentile, 75% percentile, minimum and maximum will be provided for patients with add on tenofovir (Week 24 ≥ 300 copies/ml: LDT+TDF) and patients continuing on telbivudine (Week 24 < 300 copies/ml: LDT) and overall for all scheduled post-baseline time points.

Dichotomous endpoints

Dichotomous endpoints include non-detectability of HBV DNA, ALT normalization, HBeAg loss, HBeAg seroconversion, HBsAg loss, HBV virologic breakthrough, treatment emergent HBV resistance.

For dichotomous endpoints, statistical summaries will include counts and percentages of patients with a positive response (response rate) and also 95% Confidence intervals for the response rates calculated based on (1). Separate summaries will be generated for patients with add on tenofovir (Week 24 ≥ 300 copies/ml: LDT+TDF) and patients continuing on telbivudine (Week 24 < 300 copies/ml: LDT). Summary statistics for the two groups combined will also be presented.

Time to event endpoints

Time to event endpoints include time to maintained HBV DNA non-detectability response, time to maintained HBV DNA suppression, time to virologic breakthrough, time to maintained ALT normalization.

Kaplan-Meier estimates and curves will be provided for overall patients only.

Handling of missing data (for secondary efficacy endpoints)

For the secondary efficacy endpoints, the method of handling missing data for treatment discontinuation due to efficacy is the same as what is described in Section 10.4.4. Table 10-1 shows the methods for handling missing data when patients have missing values for any of the following criteria:

- Study Discontinuation due to Treatment Failure (Clinical Disease Progression, Lack of Efficacy, or Virologic Breakthrough)
- Patient Withdrawal from the Study
- Intermittent Missing Values.

Table 10-1 Method of Handling Missing Data for Treatment Failure, Patient Withdrawal and Intermittent Missing Data

Endpoint Missing	Data Method
HBV DNA Non-detectability	
ALT Normalization	
HBV DNA Non-detectability	

This document (090095af830c511d in docbase CREDI_EH) has been digitally signed with external signatures using Entrust PKI.
Signatures manifested as of 11/5/2011 6:28:07 AM, signing status at this time: Completed (1 of 1 signatures)
Approved for report publication by Poirier Kevin in East Hanover at Sat, 05 Nov 2011 02:19:57 AM EDT

Endpoint Missing	Data Method
ALT Normalization	
HBeAg loss	
HBeAg seroconversion	
HBsAg loss	
HbsAg seroconcersion	
Serum ALT	
Serum HBV DNA	
Virologic Breakthrough	Use LOCF
Treatment emergent HBV resistance	Cumulative*

* A patient is considered to develop treatment emergent resistance at a time point if and only if he/she develops resistance prior or on that time point.

10.5.2 Safety

The assessment of safety will be based on the analyses of AEs, vital signs and laboratory evaluations. All safety analyses will be performed on safety population.

Adverse events

AEs will be coded using the MedDRA dictionary and will be summarized by presenting the number and percentage of patients having any AE, having an AE in each system organ class and having each individual AE. Furthermore, summaries by severity and relationship to study drug will be presented. All percentages will be based on the number of patients in the safety population. In the summaries, AEs will be counted only once per patient. If a patient reports the same AE more than once, it will be counted with its worst severity and closest relationship to study drug. Adverse events of special interest (AESI), include muscle weakness, myopathy or myositis are expected to be rare in this study and will be presented in a listing and described in patient narratives.

Vital signs

Vital signs will be listed and summarized over time by treatment subgroups and overall. Change from baseline will also be summarized. Notable values and changes will be tabulated.

Laboratory evaluations

Summary statistics (mean, median, standard deviation, minimum and maximum) over time will be presented for the continuous laboratory parameters. Descriptive statistics of changes from baseline by study visits will also be presented. A frequency table of results of categorical laboratory parameters will be produced. Frequency and percentage of patients with grades lab abnormality will be presented. Further more, lab abnormalities will be analyzed by shift tables and each patient is counted only once with the worst grade in the summary tables. All laboratory data will be listed with abnormal values flagged.

10.6 Sample size calculation

This is a single arm study, the sample size is not determined based on power for statistical comparison between treatment groups. The primary objective is to demonstrate the antiviral efficacy by estimating the HBV DNA non-detectability rate at Week 52. Based on the pivotal

This document (090095af830c511d in docbase CREDI_EH) has been digitally signed with external signatures using Entrust PKI.
Signatures manifested as of 11/5/2011 6:28:07 AM, signing status at this time: Completed (1 of 1 signatures)
Approved for report publication by Poirier Kevin in East Hanover at Sat, 05 Nov 2011 02:19:57 AM EDT

phase III study NV-02B-007, the estimated HBV DNA non-detectability rate is about 44% at Week 24 and 60% at Week 52 for patients treated with telbivudine mono therapy. And among the 44% patients who were HBV DNA non-detectable at Week 24, 95% of them maintained non-detectable at Week 52, while only 34% of patient who were HBV DNA detectable at Week 24 ultimately achieved non-detectability at Week 52. In this study, it is anticipated that at least 50% of the patients who do not reach undetectable HBV DNA level at Week 24 will achieve non-detectability at Week 52 by adding on Tenofovir starting from Week 24. Therefore, the overall HBV DNA non-detectability rate is estimated to be improved to at least 70%, a 10% or more improvement from the telbivudine mono therapy. With 100 patients, and under these assumptions, the estimated 95% confidence interval is 70% +/- 9%, which gives over 95% chance to show a higher HBV DNA non-detectability rate over telbivudine mono therapy in NV-02B-007 and also provides a reasonably accurate estimate.

10.7 Power for analysis of critical secondary variables

N/A

10.8 Interim analysis

There is no interim analysis planned for this study. The primary analysis will be at week 52- when all patients complete 52 weeks of treatment. The final analyses will be performed when all patients complete the study including the follow-up period.

11 Ethical considerations

11.1 Regulatory and ethical compliance

This clinical study was designed and shall be implemented and reported in accordance with the ICH Harmonized Tripartite Guidelines for Good Clinical Practice, with applicable local regulations (including European Directive 2001/20/EC, US Code of Federal Regulations Title 21, and Japanese Ministry of Health, Labor, and Welfare), and with the ethical principles laid down in the Declaration of Helsinki.

11.2 Informed consent procedures

Eligible patients may only be included in the study after providing written (witnessed, where required by law or regulation), IRB/IEC/REB-approved informed consent, or, if incapable of doing so, after such consent has been provided by a legally acceptable representative of the patient. In cases where the patient’s representative gives consent, the patient should be informed about the study to the extent possible given his/her understanding. If the patient is capable of doing so, he/she should indicate assent by personally signing and dating the written informed consent document or a separate assent form. Informed consent must be obtained before conducting any study-specific procedures (i.e. all of the procedures described in the protocol). The process of obtaining informed consent should be documented in the patient source documents.

Novartis will provide to investigators in a separate document a proposed informed consent form that complies with the ICH GCP guideline and regulatory requirements and is

This document (090095af830c511d in docbase CREDI_EH) has been digitally signed with external signatures using Entrust PKI.
Signatures manifested as of 11/5/2011 6:28:07 AM, signing status at this time: Completed (1 of 1 signatures)
Approved for report publication by Poirier Kevin in East Hanover at Sat, 05 Nov 2011 02:19:57 AM EDT

considered appropriate for this study. Any changes to the proposed consent form suggested by the investigator must be agreed to by Novartis before submission to the IRB/IEC/REB, and a copy of the approved version must be provided to the Novartis monitor after IRB/IEC/REB approval.

Women of child bearing potential should be informed that taking the study medication may involve unknown risks to the fetus if pregnancy were to occur during the study and agree that in order to participate in the study they must adhere to the contraception requirement for the duration of the study. If there is any question that the patient will not reliably comply, they should not be entered in the study.

11.3 Responsibilities of the investigator and IRB/IEC/REB

The protocol and the proposed informed consent form must be reviewed and approved by a properly constituted Institutional Review Board/Independent Ethics Committee/Research Ethics Board (IRB/IEC/REB) before study start. A signed and dated statement that the protocol and informed consent have been approved by the IRB/IEC/REB must be given to Novartis before study initiation. Prior to study start, the investigator is required to sign a protocol signature page confirming his/her agreement to conduct the study in accordance with these documents and all of the instructions and procedures found in this protocol and to give access to all relevant data and records to Novartis monitors, auditors, Novartis Clinical Quality Assurance representatives, designated agents of Novartis, IRBs/IECs/REBs, and regulatory authorities as required. If an inspection of the clinical site is requested by a regulatory authority, the investigator must inform Novartis immediately that this request has been made.

11.4 Publication of study protocol and results

Novartis assures that the key design elements of this protocol will be posted in a publicly accessible database such as clinicaltrials.gov. In addition, upon study completion and finalization of the study report the results of this trial will be either submitted for publication and/or posted in a publicly accessible database of clinical trial results.

12 Protocol adherence

Investigators ascertain they will apply due diligence to avoid protocol deviations. Under no circumstances should the investigator contact Novartis or its agents, if any, monitoring the trial to request approval of a protocol deviation, as no authorized deviations are permitted. If the investigator feels a protocol deviation would improve the conduct of the study this must be considered a protocol amendment, and unless such an amendment is agreed upon by Novartis and approved by the IRB/IEC/REB it cannot be implemented. All significant protocol deviations will be recorded and reported in the CSR.

12.1 Protocol Amendments

Any change or addition to the protocol can only be made in a written protocol amendment that must be approved by Novartis, Health Authorities where required, and the IRB/IEC/REB. Only amendments that are required for patient safety may be implemented prior to

This document (090095af830c511d in docbase CREDI_EH) has been digitally signed with external signatures using Entrust PKI.
Signatures manifested as of 11/5/2011 6:28:07 AM, signing status at this time: Completed (1 of 1 signatures)
Approved for report publication by Poirier Kevin in East Hanover at Sat, 05 Nov 2011 02:19:57 AM EDT

IRB/IEC/REB approval. Notwithstanding the need for approval of formal protocol amendments, the investigator is expected to take any immediate action required for the safety of any patient included in this study, even if this action represents a deviation from the protocol. In such cases, Novartis should be notified of this action and the IRB/IEC/REB at the study site should be informed within 10 working days.

This document (090095af830c511d in docbase CREDI_EH) has been digitally signed with external signatures using Entrust PKI.
Signatures manifested as of 11/5/2011 6:28:07 AM, signing status at this time: Completed (1 of 1 signatures)
Approved for report publication by Poirier Kevin in East Hanover at Sat, 05 Nov 2011 02:19:57 AM EDT

13 References: available upon request

Baraclude™ (entecavir) US product label (2005) Bristol-Myers Squibb Company.

Bryant ML, Bridges EG, Placidi L, et al (2001) Antiviral L-nucleosides specific for hepatitis B virus infection. Antimicrob Agents Chemotherapy; 45(1):229-35.

Chan HJY, Leung NWY, Hussain M, et al (2000) Hepatitis B antigen-negative chronic hepatitis B in Hong Kong. Hepatol; 31:763–768.

Chen, CJ, Yang HI, Su J, et al (2006) Risk of hepatocellular carcinoma across a biological gradient of serum hepatitis B virus DNA level. JAMA; 295:65-73.

Cockcroft DW and Gault MH (1976) Prediction of creatinine clearance from serum creatinine. Nephron; 16:31-41.

Di Bisceglie AM, Lai CL, Gane E, et al (2006) Telbivudine GLOBE trial: Maximal early HBV suppression is predictive of optimal two-year efficacy in nucleoside-treated hepatitis B patients. Hepatology; 44 (4 Suppl. 1):230A (abstract 112).

Epivir-HBV® (lamivudine) US product label (2004) GlaxoSmithKline.

Heathcote E, Gane E, DeMan R, et al (2007)A randomized, double-blind, companrison of tenofovir DF (TDF) versus adefovir dipivoxil (ADV) for the treatment of HBeAg positive chronic hepatitis B (CHB): Study GS-US-174-0103. Hepatology; 46: Abstract LB6.

Hepsera® (adefovir dipivoxil) US product label (2004) Gilead Sciences, Inc.

Hoofnagle JH, Doo E, Liang TJ, et al (2007) Management of Hepatitis B: Summary of a Clinical Research Workshop. N Engl J Med; 336(5):347-56.

Hoofnagle JH and di Bisceglie AM (1997) The treatment of chronic viral hepatitis. N Engl J Med; 336(5):347-56.

Janssen HLA, van Zonneveld M, Senturk H, et al. (2005) Pegylated interferon alfa-2b alone or in combination with lamivudine for HBeAg-positive chronic hepatitis B: a randomized trial. Lancet; 365:123-29.

Keeffe EB, Zeuzem S, Koff RS, et al (2007) Report of an international workshop: Roadmap for management of patients receiving oral therapy for chronic hepatitis B. Clin Gastroenterol Hepatology; 4:890-897.

Keeffe EB, Dieterich DT, Han SH, et al (2006) A treatment algorithm for the management of chronic hepatitis B virus infection in the United States: an update. Clin Gastroenterol Hepatology; 4(8):936-962.

Lai CL, Gane E, Liaw Y, et al (2005) Telbivudine (LdT) vs. lamivudine for chronic Hepatitis B: First-year results from the international phase III GLOBE trial. Slide presentation. 56th AASLD, November 11-15, 2005. San Francisco, CA.

This document (090095af830c511d in docbase CREDI_EH) has been digitally signed with external signatures using Entrust PKI.
Signatures manifested as of 11/5/2011 6:28:07 AM, signing status at this time: Completed (1 of 1 signatures)
Approved for report publication by Poirier Kevin in East Hanover at Sat, 05 Nov 2011 02:19:57 AM EDT

Lai CL, Gane E, Hsu CW, et al (2006) Two-year results from the GLOBE trial in patients with hepatitis B: greater clinical and antiviral efficacy for telbivudine (LDT) vs. lamivudine. Hepatology; 44(Suppl 1): 63A (abstract 91).

Lai CL, Leung N, Teo EK, et al (2005b) A 1-year trial of telbivudine, lamivudine, and the combination in patients with hepatitis B e antigen-positive chronic hepatitis B. Gastroenterol; 129(2):528-36.

Lee WM (1997) Hepatitis B virus infection. N Engl J Med; 337:1733-45.

Liaw YF (2005) Prevention and surveillance of hepatitis B virus-related hepatocellular carcinoma. Sem Liv Disease; 25(Suppl 1):40-47.

Lok AS, McMahon BJ(2007) AASLD Practice Guidelines-Chronic hepatitis B. Hepatology; 45(2):507-539.

Marcellin P, Chan HLY, Lai C L, et al (2007) In hepatitis B patients treated with either adefovir of telbivudine maximal early HBV suppression at 24 weeks predicts optimal one-year efficacy (abstract). Journal of Hepatology;46 (Supplement 1): Page S193.

Merican I, Guan R, Amarapuka D, et al (2000) Chronic hepatitis B virus infection in Asian countries. J Gastroentero and Hepat;15:1356-61.

Patty A, Li B, Serra I, et al (2007) In vitro anti-HBV activity of telbivudine/tenofovir and telbivudine/entecavir combinations. Hepatology; 46(Supp 1): 663A (Abstract 958).

Pegasys® (pegylated interferon alfa- 2a) US product label (2004) Roche Pharmaceuticals Inc.

Perrillo RP (1993) Interferon in the management of chronic hepatitis B. Dig Dis Sci; 38(4):577-93.

Perrillo RP, Schiff ER, Davis GL, et al (1990) A randomized, controlled trial of interferon alfa-2b alone and after prednisone withdrawal for the treatment of chronic hepatitis B. The Hepatitis Interventional Therapy Group. N Engl J Med; 323(5):295-301.

(Renault PF and Hoofnagle JH (1989)) Side effects of alpha interferon. Semin Liver Dis; 9(4):273-7.

Ristig MA, Crippin J, Aberg JA, et al (2002) Tenofovir Disporxil Fumarate Therapy for Chronic Hepatitis B in Human Immunodeficiency Virus/Hepatitis B Virus-Coinfected Individuals for Whom Interferon-alpha and Lamivudine Therapy Have Failed. J of Inf Dis;186:1844-7.

Sung JJYLJ, Zeuzem S, Chow WC, et al (2003) A randomized double-blind phase II study of lamivudine compared to lamivudine plus adefovir dipivoxil for treatment naïve patients with chronic hepatitis B: week 52 analysis. J Hepatol; 38(Supp 2) abstract 25.

(Tyzeka® (telbivudine) US product label (2006)) Idenix, Inc.

Van Bommel F, Zollner B, Sarrazin C (2006) Tenofovir for Patients with Lamivudine-Resistant Hepatitis B Virus (HBV) Infection and High HBV DNA Level During Adefovir Therapy.Hepatology,;44(2): 318-325.

This document (090095af830c511d in docbase CREDI_EH) has been digitally signed with external signatures using Entrust PKI.
Signatures manifested as of 11/5/2011 6:28:07 AM, signing status at this time: Completed (1 of 1 signatures)
Approved for report publication by Poirier Kevin in East Hanover at Sat, 05 Nov 2011 02:19:57 AM EDT

Van Bommel F, Winsche T, Mauss S, et al (2004) Comparison of Adefovir and Tenofovir in the Treatment of Lamivudine-Resistant Hepatitis B Virus Infection. Hepatology;40(6):1421-1425.

14 Appendix 1: Clinically notable laboratory values and vital signs

Please refer to site specific reference laboratory manual.

This document (090095af830c511d in docbase CREDI_EH) has been digitally signed with external signatures using Entrust PKI.
Signatures manifested as of 11/5/2011 6:28:07 AM, signing status at this time: Completed (1 of 1 signatures)
Approved for report publication by Poirier Kevin in East Hanover at Sat, 05 Nov 2011 02:19:57 AM EDT

15 Appendix 2: Prohibited Concurrent Medications/Permitted Concurrent Medication

Patients must not receive treatment with any prohibited medications during the study, unless otherwise noted. The following medications are prohibited:

1. All investigational drugs other than telbivudine or tenofovir, including (but not limited to) investigational anti-HBV drugs such as PMEA analogs (e.g., MCC-478), emtricitabine (FTC), lobucavir, entecavir, L-FMAU, L-FdFC, or other nucleoside/nucleotide HBV drug candidates, various investigational interferons, immunomodulators (e.g., IL-12, thymosin, etc).
2. All other treatments for hepatitis B, including lamivudine from other sources, alpha-interferon, and commercially available treatments indicated for conditions other than chronic hepatitis B that are being investigated to treat or may have activity against HBV (e.g.,ribavirin, famciclovir, ganciclovir, etc).
3. Prolonged use of systemic acyclovir or famciclovir defined as episodic treatment with these agents for periods exceeding 10 days every 3 months, or chronic suppressive therapy.
4. Systemic immunomodulators of any type. Proscribed therapies include, but are not limited to, IFN agents (PEG, alpha-, beta- or gamma-interferons), thymosin, interleukin-12, or other putative systemic immunomodulators.
5. Hepatotoxic drugs (e.g., dapsone, erythromycin, fluconazole, ketoconazole, rifampin or anti-tuberculosis drugs, toxic doses of acetaminophen).
6. Nephrotoxic drugs (e.g., aminoglycosides, amphotericin B, foscarnet, vancomycin, cyclosporine, tacrolimus, etc.), or frequent use of NSAIDs or aspirin (administered daily for more than one week at a scheduled dose intended for anti-inflammatory therapy).
7. Systemic corticosteroids (topical and inhaled corticosteroids are permitted).
8. Herbal medications known to cause hepatotoxicity (e.g., St. John’s Wart, milk thistle, Kava, Jin Bu Huan, Yuzhitang, germander, chaparral, shark cartilage, mistletoe, slim 10, Lipokinetix, etc.).
9. Alcohol or illicit drug abuse. For the purposes of the present study, alcohol abuse is arbitrarily defined as frequent consumption of alcoholic beverages with an average daily intake of more than 40g of ethanol.

If medically necessary, treatment with non-hepatotoxic medications is allowed. However, new medications for study patients should be initiated with caution, since they may increase the possibility of adverse events.

Herbal medications will be allowed except for those that cause known hepatotoxicity. All herbal medications will be captured on the concurrent medication eCRF as “herbal supplement” with the indication for use documented.

This document (090095af830c511d in docbase CREDI_EH) has been digitally signed with external signatures using Entrust PKI.
Signatures manifested as of 11/5/2011 6:28:07 AM, signing status at this time: Completed (1 of 1 signatures)
Approved for report publication by Poirier Kevin in East Hanover at Sat, 05 Nov 2011 02:19:57 AM EDT

16 Appendix 3: Initial Muscle Symptom Questionnaire

Muscle Symptom Questionnaire

1. Do you have *new or worsening** difficulty rising from sitting positions (for example, rising from a chair, toilet, getting out of a car)? If yes**, when did you first notice this?

2. Do you have *new or worsening** difficulty raising upper extremities (for example, brushing or combing hair, reaching for things above the shoulders, pulling or pushing objects)? If yes**, when did you first notice this?

3. Do you have *new or worsening** difficulty rising from lying positions to sitting positions (for example, getting up from bed, sofa, chaise lounge)? If yes**, when did you first notice this?

4. Do you have *new or worsening** muscle pain in one or more limbs at rest or after exercise? If yes**, when did you first notice this?

5. Do you have *new or worsening** difficulties with walking, running or exercising? If yes**, when did you first notice this?

6. Do you have any other musculoskeletal complaints? If yes**, specify. When did you first notice this?

**New or worsening* = since starting study drug treatment for chronic hepatitis B

**If any question is answered 'yes', please enter the appropriate adverse event (AE) and information regarding the AE in the Case Report Form (CRF). The following information should be collected at subsequent visits, and until stabilization or resolution of these AE.

This document (090095af830c511d in docbase CREDI_EH) has been digitally signed with external signatures using Entrust PKI.
Signatures manifested as of 11/5/2011 6:28:07 AM, signing status at this time: Completed (1 of 1 signatures)
Approved for report publication by Poirier Kevin in East Hanover at Sat, 05 Nov 2011 02:19:57 AM EDT

17 Appendix 4: Follow up Muscle Symptom Questionnaire

Follow-up to Muscle Symptom Questionnaire

1. Has there been any change (improvement or worsening), or no change, since the last visit when rising from sitting positions? When did the change occur? If resolved, when did it resolve?

2. Has there been any change (improvement or worsening), or no change, since the last visit when raising upper extremities? When did the change occur? If resolved, when did it resolve?

3. Has there been any change (improvement or worsening), or no change, since the last visit when rising from lying positions to sitting positions? When did the change occur? If resolved, when did it resolve?

4. Has there been any change (improvement or worsening), or no change, since the last visit in muscle pain in one or more limbs at rest or after exercise? When did the change occur? If resolved, when did it resolve?

5. Has there been any change (improvement or worsening), or no change, since the last visit in the difficulties you experienced with walking, running or exercising? When did the change occur? If resolved, when did it resolve?

6. Has there been any change (improvement or worsening), or no change, since the last visit in any other musculoskeletal complaints? When did the change occur? If resolved, when did it resolve?

This document (090095af830c511d in docbase CREDI_EH) has been digitally signed with external signatures using Entrust PKI.
Signatures manifested as of 11/5/2011 6:28:07 AM, signing status at this time: Completed (1 of 1 signatures)
Approved for report publication by Poirier Kevin in East Hanover at Sat, 05 Nov 2011 02:19:57 AM EDT

18 Appendix 5: Muscle Strength Testing Exam

Neuromuscular Exam with Strength Testing

Was a neuromuscular exam with strength testing performed? ***No*** ☐ ***Yes**** ☐

****If YES, please provide results.***

**Body Location** (Enter only one code per line)	**Specify Muscle Group** (Enter only one major muscle group per line)	**Left or Right** (L or R)	***Strength Score (0 - 5)***

Body Location	***Strength Score***
A = Arm	***0 = no contraction***
L = Leg	***1 = muscle flicker, but no movement***
T = Trunk	***2 = movement possible, but not against gravity (test the joint in its horizontal plane)***
H = Head	***3 = movement possible against gravity, but not against resistance by the examiner***
O = Other	***4 = movement possible against some resistance by the examiner***
	5 = normal strength

This document (090095af830c511d in docbase CREDI_EH) has been digitally signed with external signatures using Entrust PKI.
Signatures manifested as of 11/5/2011 6:28:07 AM, signing status at this time: Completed (1 of 1 signatures)
Approved for report publication by Poirier Kevin in East Hanover at Sat, 05 Nov 2011 02:19:57 AM EDT
